# FoxP3 expression by retinal pigment epithelial cells: transcription factor with potential relevance for the pathology of age-related macular degeneration

**DOI:** 10.1186/s12974-022-02620-w

**Published:** 2022-10-22

**Authors:** Ahmad Samir Alfaar, Lucas Stürzbecher, Maria Diedrichs-Möhring, Marion Lam, Christophe Roubeix, Julia Ritter, Kathrin Schumann, Balasubramaniam Annamalai, Inga-Marie Pompös, Bärbel Rohrer, Florian Sennlaub, Nadine Reichhart, Gerhild Wildner, Olaf Strauß

**Affiliations:** 1 Experimental Ophthalmology, Department of Ophthalmology, Charité - Universitätsmedizin Berlin, Corporate Member of Freie Universität, Berlin Institute of Health, Humboldt-University, 10117 Berlin, Germany; 2 Department of Ophthalmology, University Hospital of Ulm, 89075 Ulm, Germany; 3 Section of Immunobiology, Department of Ophthalmology, University Hospital, LMU Munich, 80336 Munich, Germany; 4 Institut de La Vision, Sorbonne Université, INSERM, CNRS, 75012 Paris, France; 5 Institut Für Med. Mikrobiologie, Immunologie Und Hygiene, TU München, 81675 Munich, Germany; 6 Department of Ophthalmology, College of Medicine, Medical University South Carolina, Charleston, SC 29425 USA

**Keywords:** IL-1β, Ca-channels, CRISPR/Cas9, Phosphorylation, RPE, Age-related macular degeneration, FoxP3, Immune barrier, Immune privilege of the retina

## Abstract

**Background:**

Forkhead-Box-Protein P3 (FoxP3) is a transcription factor and marker of regulatory T cells, converting naive T cells into Tregs that can downregulate the effector function of other T cells. We previously detected the expression of FoxP3 in retinal pigment epithelial (RPE) cells, forming the outer blood–retina barrier of the immune privileged eye.

**Methods:**

We investigated the expression, subcellular localization, and phosphorylation of FoxP3 in RPE cells in vivo and in vitro after treatment with various stressors including age, retinal laser burn, autoimmune inflammation, exposure to cigarette smoke, in addition of IL-1β and mechanical cell monolayer destruction. Eye tissue from humans, mouse models of retinal degeneration and rats, and ARPE-19, a human RPE cell line for in vitro experiments, underwent immunohistochemical, immunofluorescence staining, and PCR or immunoblot analysis to determine the intracellular localization and phosphorylation of FoxP3. Cytokine expression of stressed cultured RPE cells was investigated by multiplex bead analysis. Depletion of the FoxP3 gene was performed with CRISPR/Cas9 editing.

**Results:**

RPE in vivo displayed increased nuclear FoxP3-expression with increases in age and inflammation, long-term exposure of mice to cigarette smoke, or after laser burn injury. The human RPE cell line ARPE-19 constitutively expressed nuclear FoxP3 under non-confluent culture conditions, representing a regulatory phenotype under chronic stress. Confluently grown cells expressed cytosolic FoxP3 that was translocated to the nucleus after treatment with IL-1β to imitate activated macrophages or after mechanical destruction of the monolayer. Moreover, with depletion of FoxP3, but not of a control gene, by CRISPR/Cas9 gene editing decreased stress resistance of RPE cells.

**Conclusion:**

Our data suggest that FoxP3 is upregulated by age and under cellular stress and might be important for RPE function.

**Supplementary Information:**

The online version contains supplementary material available at 10.1186/s12974-022-02620-w.

## Background

The immune privilege of the eye, by which inflammatory responses are limited, is maintained by different mechanisms, including multiple blood–eye barriers [1–4]. Three different blood–eye barriers exclude non-activated immune cells from the entry of the eye or educate the invading immune cells to switch to a tolerant/repairing phenotype. Shechter et al. [4] further classified these barriers into two types: “true blood barriers” or “blood–aqueous barriers”. The so-called “true blood barrier”, the inner retinal barrier, is formed by the retinal blood vessels equipped with tight junctions of the endothelial cells and surrounded by pericytes and Müller cells, which aim at sealing the retina against invasion from the bloodstream. The two other barriers are the blood–aqueous barrier of the non-pigmented ciliary body epithelium and the outer blood–retina barrier, or the retinal pigment epithelium (RPE) [5–8]. These barriers are called “educational” or “regulating gates” and consist of fenestrated endothelia that face the ocular epithelia with tight junctions. Those epithelia interact with invading cells of the immune system by secreting immunosuppressive or immunoregulatory factors and expressing surface molecules with immunoregulatory or cytotoxic functions like FasL and PDL1. Potentially destructive effector cells of both the innate and the adaptive arm of the immune system are converted to a regulatory or repairing phenotype when they pass these barriers [4].

When activated cells of the immune system interact with or pass through the RPE, its phenotype changes from maintaining tolerance to immune stimulation [9–15]. To efficiently interact with the immune system, the RPE expresses many immune surface receptors/ligands, including anaphylatoxin receptors [16–26], Toll-like receptors [27–32], and receptors for cytokines like IL-1β receptor [17, 33–35] or TNF-α receptor [12, 14, 28, 36–44]. Furthermore, the RPE can secrete immune-relevant cytokines and chemokines like MCP-1/CCL2 [15, 16, 45–49], IL-6 [27–29, 31, 47, 49, 50] or IL-12, IFN-γ, IL-8/CXCL8 and IP-10/CXCL10 [49], as well as complement factors such as C3, C5 and CFH [16, 19, 21, 39, 51–58]. Interestingly, cytokines and chemokines released from macrophages or T cells can induce the secretion of VEGF-A in RPE cells, indicating that an interaction with the immune system might also promote an angiogenic phenotype [12–14, 33, 35, 57]. Studies from both animal and in vitro models provide examples of the dynamic interplay between the RPE and immune cells. Invading mononuclear phagocytes secrete TNF-α, IL-6, and IL-1β, which in turn provoke pro-inflammatory reactions by the RPE [12, 14, 37–41]. The RPE secretes MCP-1/CCL2 and complement factors that attract monocytes and activate the monocyte pro-inflammatory phenotype M1. The first step towards the shift to immune activation might be monocytes that escape control by the RPE [12], activation of inflammasomes in stressed RPE, and the RPE’s interaction with an overactive complement system [17, 36, 59], explaining the association of the risk for the development of age-related macular degeneration (AMD) with polymorphisms in complement genes [16, 23, 53, 59]. The detailed mechanisms that induce the switch in the RPE-phenotype from immune inhibitory to inflammatory and/or angiogenic are so far unknown, but represent major targets in therapy development.

Surprisingly, we have found that the RPE can express the transcription factor FoxP3 (forkhead box P3), which was previously thought to be specifically expressed by regulatory T cells [16, 60, 61]. Human and rat effector T cells transiently express FoxP3 after activation. FoxP3 expression is maintained when those cells differentiate into regulatory cells (Tregs) but is downregulated if the cells keep their effector phenotype [62, 63]. While FoxP3 is localized in the cytoplasm of activated effector cells, in Tregs it is translocated to the nucleus [64]. FoxP3-positive Treg cells can suppress inflammation and promote regeneration and tissue homeostasis [65–67]. The phenotypes of the FoxP3-positive regulatory cells depend on the level of FoxP3 expression: resting Tregs express lower levels, while effector Tregs have higher levels of FoxP3. Furthermore, the impact of FoxP3 on cell function depends on splicing variants and several post-translational modifications like methylation, acetylation, prenylation, ubiquitination, glycosylation, and phosphorylation that modify and regulate the function of FoxP3 [65].

Cell types other than Tregs that can express FoxP3 have been identified, such as epithelial cells of the prostate [68], ovary [69], and mammary glands [70], as well as a variety of cancer cells [71–73]. While prostate, ovary, and mammary glands are important for reproduction and are thus immune privileged, cancer cells often take advantage of tolerance-inducing mechanisms to protect themselves from immune attacks. Several observations in an earlier study indicate a role of FoxP3 expression in the RPE that is associated with local immune reactions. Normally, the RPE does not express FoxP3 but rather initiates its expression under conditions of stress, as occurs in experimental uveitis [16]. Furthermore, stimulation of RPE cells with anaphylatoxins leads to increased expression of complement factors/receptors as well as the secretion of cytokines such as MCP-1/CCL2, VEGF-A, and IL-6 that are associated with increased FoxP3 phosphorylation [16]. Oxidative stress, one of the risk factors promoting AMD, also induces FoxP3 expression in the RPE [59]. Thus, FoxP3 could be regarded as a general immune regulatory transcription factor in the RPE and might therefore play an essential role in the development of diseases such as AMD.

While the role of FoxP3 in T lymphocyte function and regulation has been extensively investigated, its role in the RPE function remains unexplored. By analogy to FoxP3 + Tregs that play a critical role in immune tolerance and chronic stress, which promotes a pro-inflammatory phenotype, we propose that FoxP3 induces regulatory functions of the RPE as a part of its role in maintaining the outer blood–retina barrier function. FoxP3 thus might play a major role for RPE function, as prior evidence demonstrates that its expression in the RPE is stress- and disease-related. The aim of this study was to test this hypothesis by analyzing the FoxP3 expression both in vivo and in vitro AMD models, including human and animal tissue, and in RPE cell cultures by application of IL-1β.

## Material and methods

### Animals

All animal experiments were conducted in accordance with the ARVO statement for the use of animals in ophthalmic and vision research. Mouse experiments were approved by the LaGeSo under G0039/19, except for the smoke exposure experiment which was approved by the IACUC at MUSC under institutional approval number 00399. We used C57BL/6J mice for laser-induced choroidal neovascularization (CNV) and as controls for the *Cx3cr1*^*GFP/GFP*^ mice that are on the C57BL/6 J genetic background. *Cx3cr1*^*GFP/GFP*^ mice older than 10 months are hyperinflammatory and develop age-dependent subretinal inflammation, a hallmark of AMD [74]. The animals were kept under a 12-h light–dark cycle, standard environmental conditions, and food and water were provided ad libitum.

The laser-induction of CNV was performed as previously described [75]. In brief, four laser burns were applied around the optic nerve using an Argon laser (120 mW, 100 ms, 50 µm), under anesthesia with 1% ketamine hydrochloride (Actavis, Munich, Germany) and 0.1% xylazine (Rompun; Bayer Vital GmbH, Leverkusen, Germany), and dilated pupils (2.5% phenylephrine-hydrochloride and 0.5% tropicamide (Charité Apotheke, Berlin, Germany). Retinal bleeding after laser burn led to exclusion of the eye.

Experimental autoimmune uveitis was induced in Lewis rats as previously described [49] and was approved by the government of Upper Bavaria (ROB-55.2–1-2532.Vet_02-15–225). In brief, Lewis rats were subcutaneously immunized with retinal S-antigen peptide PDSAg (Polypeptide Laboratories, Strasbourg, France) in CFA (BD Biosciences, Heidelberg, Germany), the experiment was terminated 30 days later, and eyes were collected for cryosections.

### Immunohistochemistry of RPE/choroid flatmounts

RPE/choroid flatmounts from mouse eyes were prepared as previously described [75]. In short, eyes were enucleated, fixed in 4% paraformaldehyde for 12 min at room temperature, and sectioned at the limbus; the retinas were removed from the RPE/choroid/sclera and 6–8 radial sections were made. After incubation of the RPE/choroid/sclera tissue in 5% Triton X-100 in TBS overnight at 4 °C, the samples were incubated in blocking buffer for 1 h (5% BSA in TBS). Subsequently, an incubation with rabbit polyclonal anti-FoxP3 (Novus, 1:200) antibody and ActiStain555-conjugated phalloidin (Biomol, Hamburg, Germany, 1:500) followed for 48 h. Rat anti-mouse CD102 (BD-Pharmingen, Heidelberg, Germany, 1:200) was used to visualize the laser scars. After a few washes, the samples were incubated for 1 h at room temperature with the appropriate Alexa Fluor®-conjugated secondary antibodies (Thermo Fisher Scientific; 1:1000). Tissues were embedded in DAKO fluorescence mounting medium (Agilent, Ratingen, Germany) and images were taken using a LSM 510 confocal laser-scanning microscope (Zeiss, Jena, Germany) and digitalized using ZEN 3.1 Blue Edition software (Zeiss, Oberkochen, Germany).

### Immunohistochemistry of sagittal sections

#### Human AMD samples

Eyes from donors with a known history of AMD and age-matched controls were collected from the Minnesota Lions Eye Bank. We examined 4 eyes of the group “Non-Geographic Atrophy” (Non-GA) and 3 eyes with “Geographic Atrophy” (GA). The group of Non-GA included three males and one female, the mean age was 85.75 ± 1 years, and the time of death-to enucleation was 193.75 ± 9 min (3:14 h). The death causes in the Non-GA were intracerebral hemorrhage, cardiac arrest, lung cancer, and dementia. The group of GA included two females and one male, the mean age was 85 ± 2.6 years, and the death-to-enucleation time was 278.5 ± 60 min (4:38 h). The death causes in the GA group were breast cancer, congestive heart failure, and an acute cardiac event. When reported, the death-to-cooling times in both groups were between 1 and 2:25 h. The posterior segment was fixed for 4 h in 4% PFA, dissected, embedded in paraffin, and sectioned. Slides were generously sent to us for further processing. Horse serum was used to block unspecific binding. The primary antibody, rabbit polyclonal FoxP3 (1:200, Novus Biologicals; the same antibody as used for ARPE-19 staining below) was incubated with the sections at 4 °C overnight. A secondary AP-coupled anti-rabbit antibody was incubated for 1 h at RT and a Fast Red substrate kit (Sigmafast Fast Red TR/Naphthol AS-MX, Sigma/Merck) was used to visualize positive staining under brightfield microscopy using a DM5500 microscope (Leica, Nanterre, France).

#### Rat eyes

Eyes from killed animals were embedded in Tissue Tec OCT compound (Paesel and Lorey, Frankfurt/Main, Germany) and immediately snap frozen in methyl butane (Merck, Darmstadt Germany) at − 70 °C to avoid shrinking of the tissue. Air-dried cryosections (8 µm, performed with a CryoStat Microm HM560 Microtom, Thermo Scientific, Dreieich, Germany) were fixed in ice-cold acetone for 10 min and then dried, and the sections were incubated with rabbit anti-rat FoxP3 antibody (Novus Biologicals, Abingdon, UK) diluted 1:100 in PBS/3% donkey serum overnight at 4 °C in a humid chamber. After washing 3× with PBS, Cy3-conjugated affinipure donkey anti-rabbit IgG(H + L) (Jackson Laboratories, Dianova, Hamburg, Germany) was added as a secondary antibody at a dilution of 1:100 in PBS and incubated for 1 h at RT in the dark. Then the slides were washed 3× with PBS and mounted with Vectashield HardSet with DAPI H-1500 (Biozol, Eching, Germany). Pictures were taken with an Axio Observer 7 with ApoTome (Zeiss, Oberkochen, Germany).

### Cell culture

The research team of Prof. Marius Ader (Center of Regenerative Medicine, Dresden Germany) provided human RPE cells differentiated from inducible stem cells (iPS-RPE). iPS-RPE  cells (differentiated from the iPS cell line CRTDi004-A; CTRD Dresden registered under https://hpscreg.eu/cell-line/CRTDi004-A) were grown on filter inserts and reached transepithelial resistance at 600 Ωcm^2^. The cells were maintained in mTeSR™ plus medium (Stemcell Technologies, Cologne Germany) at 37 °C and 5% CO_2_. The local Ethics Committee approved the use of human material under the registration number EA1/024/17.

ARPE-19 cells were cultured in DMEM/F12 (Thermo Fisher, Darmstadt, Germany) with Glutamax (stable glutamine) supplemented with 10% FCS and 50 U penicillin/50 mg streptomycin at 37 °C and 5% CO_2_. The cell density differed between confluent and non-confluent (50–70% confluency), with confluent cells forming a closed monolayer. Before any treatment, medium was exchanged with serum-free medium for 24 h. ARPE-19 cells were then incubated with 100 ng/ml IL-1β (Sigma Aldrich/Merck, Darmstadt, Germany) for 6 min, 1 h, and 2 h, respectively, at 37 °C and 5% CO_2_, supernatants were collected, and cells immediately harvested. Confluent cells had a density of 30.000 cells per cm^2^, non-confluent cultures 20.000 cells per cm^2^.

### Generation and characterization of APRE-19 KO cells

Electroporation of ARPE-19 cells was performed using the SF Cell Line EP Kit (Lonza) and 4D-Nucleofector (Lonza; nucleofection code: DN-100). Each reaction contained 5 × 10^5^ ARPE-19 cells, (grown confluently or non-confluently and trypsinized immediately prior to electroporation) 4 μl of the respective Cas9 RNP and 1 μl of electroporation enhancer (IDT). Cas9 RNPs were assembled with 100 μM crRNA (IDT) and 100 μM tracrRNA (IDT) mixed in a 1:1 ratio and incubated for 5 min at 96 °C to generate 50 μM crRNA–tracrRNA duplexes. Then, 40 μM S. pyogenes Cas9-NLS (purified using published protocols [76]; Cas9 expression plasmid: pMJ915; Addgene #69090, Watertown, MA, USA) was slowly added to the crRNA–tracrRNA duplexes and incubated for 15 min at room temperature. The crRNAs targeting CXCR4 and FoxP3 have been functionally validated in human T cells before with editing efficiencies of 60–90% or 60–80%, respectively [77, 78]; protospacer sequences: FoxP3: TCATGGCTGGGCTCTCCAGG; CXCR4: GAAGCGTGATGACAAAGAGG; non-targeting control: GGTTCTTGACTACCGTAATT. After nucleofection, 80 μl pre-warmed DMEM + 10% FCS was added and cells rested for 10 min at 37 °C. Transfected ARPE-19 cells were seeded again to grow to either 100% or 50% confluency in 6 well plates. 1, 3 and 10 days after nucleofection DNA was isolated from all conditions using QuickExtract (Epicenter) according to the manufacturer`s recommendation. The editing efficiencies were determined by amplicon sequencing followed by Sanger sequencing. Each PCR reaction contained 12.5 μl of 2 × iProof HF master mix (Bio-Rad), 1.25 μl of 10 μM forward primer (Sigma), 1.25 μl of 10 μM reverse primer (Sigma), and 9 μl of H_2_O (primer sequences: CXCR4 fwd: AGAGGAGTTAGCCAAGATGTGACTTTG, CXCR4 rev: GGACAGGATGACAATACCAGGCAGGATAAGGCC, FoxP3 fwd2: AGCTCTGCAACTTATTAGCTG, FoxP3 rev: GCTTAAAGACGGCCATTC). The thermocycler setting consisted of one step at 95 °C for 3 min, followed by 35 cycles at 94 °C for 20 s, 65 °C for 20 s, and 72 °C for 1 min (wherein the annealing temperature was decreased by 0.5 °C per cycle); followed by 25 cycles at 98 °C for 20 s, 58 °C for 20 s, and 72 °C for 1 min. Sanger sequencing was performed by Microsynth AG (Balgach, Switzerland). Sequencing traces were analyzed with the TIDE webtool (http://tide.nki.nl; [79]).

### Calcium imaging

ARPE-19 cells grown on 15 mm glass cover slips (8.5 × 10^3^ cells · cm^−2^) were kept under serum-free conditions overnight. Calcium imaging was carried out as previously described in [16]. In short, after incubation for 40 min with fura-2/AM (2 µM, Invitrogen), the cover slips were placed in a custom-made recording chamber (bath solution consisting of (in mM): 138 NaCl, 5.8 KCl, 0.41 MgSO_4_, 0.48 MgCl_2_, 0.95 CaCl_2_, 4.17 NaHCO_3_, 1.1 NaH_2_PO_4_, 25 HEPES) and imaged using a Zeiss Axiovert 40 CFL inverted microscope (Carl Zeiss AG) equipped with a 40 × oil immersion objective, a Visichrome High Speed Polychromator System (Visitron Systems), and a high-resolution CCD camera (CoolSNAP EZ, Photometrics). IL-1β (100 ng/ml) was added in the presence of the following agonists/blockers: (R)-(+)-BayK 8644 (10 µM, Tocris, Wiesbaden, Germany), Thapsigargin (1 µM, Acros, Schwerte, Germany), Ruthenium Red (1 µM, Alomone, Jerusalem, Israel), and Dantrolene (1 µM), LY294002 (50 µM; Cayman Chemical Tallinn, Estonia). Fura-2/AM signal was acquired using the MetaFluor Fluorescence Ratio Imaging Software (Visitron Systems). Fluorescence intensity of Fura-2 was detected at an emission wavelength of 505 nm, while the excitation wavelengths were set to 340/380 nm. Changes in intracellular free Ca^2+^ are presented as changes of the ratios of the fluorescence of the two excitation wavelengths (dF/F) in relation to the baseline (ddF/F).

### Immunofluorescence staining of ARPE-19 cells after stimulation with IL-1β

Cells grown on 15-mm-diameter cover slips to confluence or non-confluence were fixed in 4% PFA for 10 min and permeabilized in 5% Triton X-100 in TBS for 10 min, or alternatively fixed for 15 min with ice-cold methanol. After blocking in 5% BSA in TBS for 30 min, the primary antibody (rabbit polyclonal FoxP3, Novus Biologicals, 1:200) was applied overnight at 4 °C. After incubation with an appropriate secondary antibody conjugated with AF647 or Cy3 for 1 h at RT, nuclei were counterstained with DAPI (according to supplier; Sigma, Taufkirchen, Germany). Coverslips were mounted in fluorescent mounting Medium (DAKO, Glastrup, Denmark) or Entellan (Merck, Darmstadt, Germany) and visualized using an LSM 510 confocal laser-scanning microscope (Zeiss, Jena, Germany) and ZEN software 3.1 Blue Edition (Zeiss, Oberkochen, Germany) or a Zeiss Axioskop 2plus an Axio Observer 7 with ApoTome (Zeiss, Oberkochen, Germany) or imaged with a Zeiss Axioskop 2plus (Carl Zeiss, Jena, Germany), and photographs were taken with a Sony CyberShot DSC-S70 3.3 mp digital camera. (Carl Zeiss, Jena, Germany). The integrated density of pixels in the nucleus was determined using ImageJ software [80].

### Dot blotting

ARPE-19 cells were grown on Transwell plates as 4-week-old monolayers or as subconfluent cells (80% confluency) on regular plates. The cultures of each plate were switched to serum-free medium overnight prior to the experiment. Cells were stimulated with 100 ng/ml IL-1β (Sigma Aldrich) or equal amounts of vehicle (PBS) for 1 h, followed by careful washing with ice-cold PBS 3 times and harvesting in sucrose isolation buffer (250 mM sucrose, 1 mM EGTA, 10 mM HEPES, and 1 mg/ml fatty acid-free BSA; pH of 7.4). Cells were homogenized using a Dounce homogenizer and centrifuged at 700*g* for 5 min to obtain supernatant (cytosol) and pellet (nuclear fraction). The supernatant was collected in phosphatase inhibitors (1:100), 1 mM sodium orthovanadate, 1 mM sodium fluoride, Triton X-100 and 4% SDS. The pellet was washed twice with isolation buffer and centrifuged at 1000*g* for 5 min. The pellet/nuclear fraction was resuspended in RIPA buffer containing 50 mM Tris·HCl, 150 mM NaCl, 0.1% SDS, 0.5% sodium deoxycholate, and 1% Triton X-100 (pH 7.4) with phosphatase inhibitors (1:100), 1 mM sodium orthovanadate, and 1 mM sodium fluoride. Dot blotting was performed as described by us previously [81]. In short, equal amounts of protein (1.5 µg) were loaded per fraction on a 96-well plate (Bio-Dot® Microfiltration Apparatus; Bio-Rad Laboratories Inc.) and vacuum transferred onto nitrocellulose membranes. Membranes were incubated with primary antibodies (1:1000) against Phospho-FoxP3 (Ser 418; Abgent Biotech) or FoxP3 (Cell Signaling Technologies) overnight. Antibodies against GAPDH (cytoplasm; Cell Signaling Technologies) and histone H3 (nuclear fraction; Cell Signaling Technologies) were used for normalization. Proteins were visualized with horseradish peroxidase-conjugated secondary antibodies (Santa Cruz Biotechnology) followed by incubation with Clarity™ Western ECL Blotting Substrate (Bio-Rad Laboratories, Inc.) and chemiluminescent detection. Protein dots were scanned and densities quantified using ImageJ software [80].

### RNA isolation, cDNA synthesis and RT-PCR

For murine samples retina and RPE were harvested and immediately stored in liquid N2. The preparation technique yields RPE and choroid. The tissue was homogenized with Qiazol Lysis Reagent (Qiagen) and Precellys ceramic beads (Peqlab Biotechnology, Erlangen, Germany). Total RNA was isolated using a RNeasy Mini Kit (Qiagen). After reverse transcription with the High-Capacity cDNA Reverse Transcription Kit (Applied Biosystems, Waltham, USA), RT-qPCR was performed using the QuantStudio 3 Real-Time PCR System (Applied Biosystems) with TaqMan Fast Universal PCR Master Mix (Scientific, Waltham, MA, USA). Primer and probes were purchased from Thermo Fisher (Schwerte, Germany) or designed using Primer Express 3.0 and synthesized by BioTez (Germany) (see Table 1). The target mRNA expression was quantitatively analyzed with the standard curve method. All expression values were normalized to the housekeeping gene 18S rRNA.

**Table 1:** Mouse PCR primers

Gene	Company	Assay ID	Forward (5’- 3’)	Reverse (5’- 3’)	Probe
CXCL1	BioTeZ Berlin-Buch GmbH		CTG CAC CCA AAC CGA AGT C	AGC TTC AGG GTC AAG GCA AG	
MCP-1	BioTeZ Berlin-Buch GmbH		GGC TCA GCC AGA TGC AGT TAA	CCT ACT CAT TGG GAT CAT CTT GCT	CCC CAC TCA CCT GCT GCT ACT CAT TCA
IL-1β	Thermo Fisher	Mm00434288			
18S	BioTeZ Berlin-Buch GmbH		ACA TCC AAG GAA GGC AGC AG	TTT TCG TCA CTA CCT CCC CG	CGC GCA AAT TAC CCA CTC CCG AC

### Cytokine/chemokine secretion

Supernatants from confluent and subconfluent ARPE-19 cell cultures, without exchange of medium (DMEM with 5% FCS and stable glutamine) for 2 weeks were tested. In some experiments, a pipet scratch 24 h prior to the experiment induced further mechanical stress to cells. Cytokine secretion after treatment with 100 ng recombinant human IL-1β/ml medium (OriGene, Rockville, USA) was measured in supernatants collected and frozen after 6 min, 1 h and 2 h of incubation with IL-1β, at which point the cells were harvested and immediately shock frozen at -80 °C. Immediately after thawing, the supernatants were tested by human Bio-Plex bead analysis (Bio-Rad Laboratories Inc., Hercules, USA) according to the manufacturer's instruction. Tested analytes were IL-1α IL-1β, IL-1ra, IL-6, IL-8/CXCL8, IL-10, IL-12 (p70 and p40), IFN-γ, MCP-1/CCL2, MCP-3/CCL7, PDGF, IL-17, IL-13, and VEGF, but only those that were secreted by the ARPE-19 cells are shown. The final values obtained in the bioplex analysis were calculated from the median value of fluorescence of at least 50 measured beads per analyte and sample.

### Data analysis

All data are presented as mean values ± SEM. Statistical significance was calculated using the Mann–Whitney U test for Ca^2+^-Imaging analyses and protein secretion analyses. For immunocytochemistry, western blot, and gene expression analyses, Student’s t-test was used (p values *p < 0.05, **p < 0.01, and ***p < 0.001). All calculations were performed in Graph Pad Prism (Version 9.3.1), Sigma Plot 14.0 (Systat, San Jose, USA), and Excel 2016.

## Results

Our previously published work showed that under normal conditions in vivo the RPE does not express FoxP3, while it does upregulate FoxP3-expression in uveitis [16], suggesting a correlation with stress situations like ocular inflammation. To test this hypothesis, we investigated FoxP3-expression in the context of AMD, a retinal disease that combines the risk factors of age and polymorphisms in genes of the innate immune system, and which is proposed to be caused by oxidative stress and chronic, low-grade inflammation as major pathomechanisms. Thus, we investigated RPE cells for FoxP3 expression under the conditions of age and AMD-relevant pathologic scenarios in various species, including humans.

**Fig. 1:**
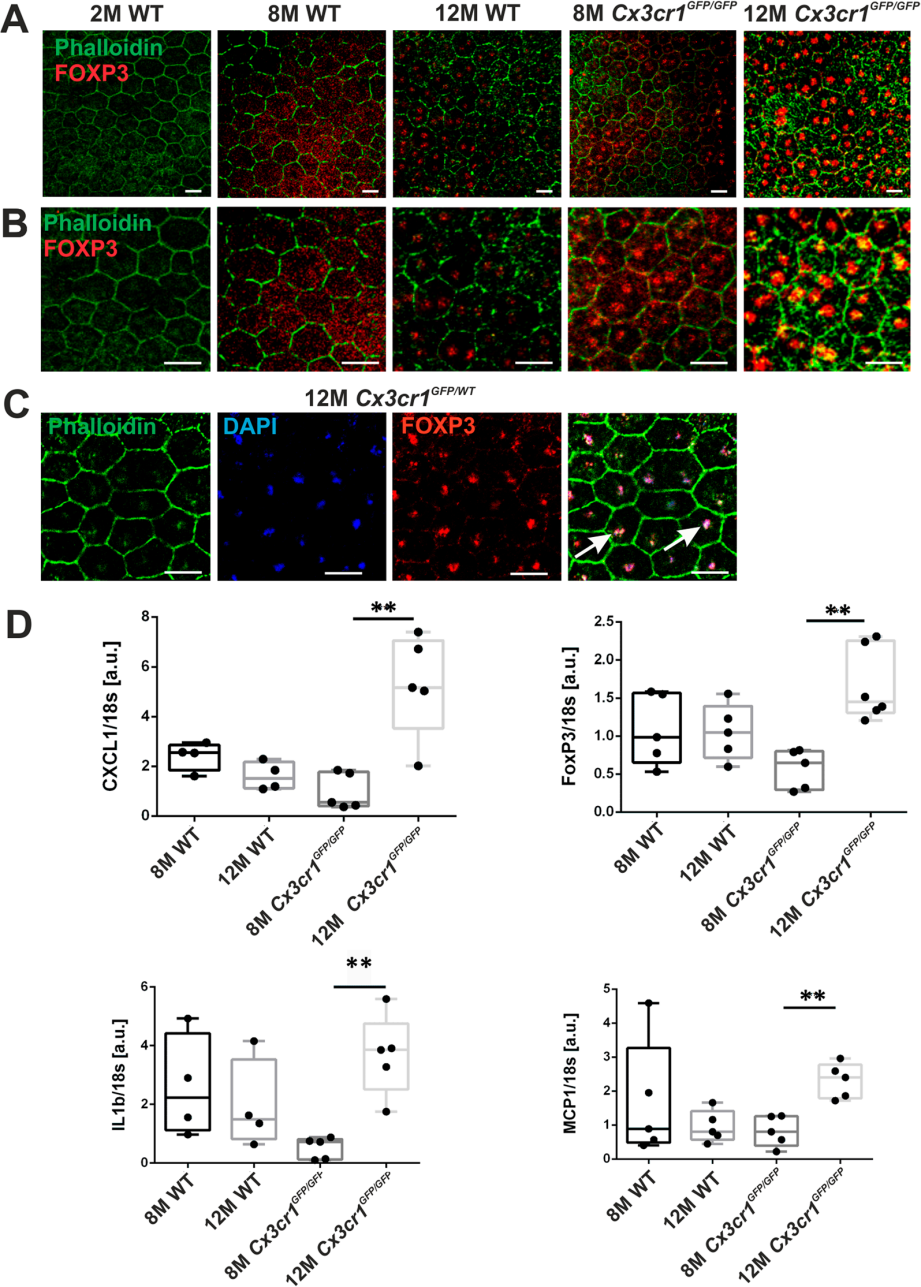
FoxP3 and cytokine expression in *Cx3cr1*^*GFP/GFP*^ mice. **A** RPE flatmount preparations from transgenic mice that display features of geographic atrophy (*Cx3cr1*^*GFP/GFP*^ mice and their wild-type littermates) stained with phalloidin (green) and for FoxP3 (red) at different ages: panels from left to right: 2, 8, 12 month wild type (WT) and 8 and 12 month *Cx3cr1*^*GFP/GFP*^ mice. Scale bar represents 20 µm. **B** Same as A, but at higher magnification to highlight the subcellular localization of FoxP3. Scale bar represents 20 µm. **C** Verification of FoxP3 localization in the nucleus: RPE flatmounts from 12-month *Cx3cr1*^*GFP/WT*^ mice were stained (from left to right) with phalloidin (green), with DAPI (blue) and for FoxP3 (red); the red dots clearly indicate the presence of FoxP3 in nucleus. Scale bar represents 20 µm. **D** Changes in gene expression of the cytokines IL-1β, MCP-1, CXCL1 and the transcription factor FoxP3 in the RPE/choroid of *Cx3cr1*^*GFP/GFP*^ mice and their wildtypes in arbitrary units (a.u.), graphs arranged to compare the progression from the ages 8 months to 12 months (normalized to 18S RNA). (CXCL1 = chemokine (CXC-motif) ligand-1; IL-1β (or IL1b) = interleukin-1β; FoxP3 = forkhead box protein P3; MCP-1 = monocyte chemoattractant protein). Student’s t-test was performed, p values **p < 0.01; *N* = 5

In the first set of experiments, we investigated the expression of FoxP3 in the RPE of rodent models (Fig. 1). The *Cx3cr1*^*GFP/GPF*^ mouse lacks the expression of the fractalkine receptor Cx3cr1, which causes a subsequent hyperinflammatory phenotype. These mice develop age-dependent subretinal inflammation, a hallmark for AMD [11, 15, 74]. The model combines aging, an inflammatory response with invading monocytes, and loss of RPE cells. We stained the RPE of RPE/choroidal flatmount preparations from *Cx3cr1*^*GFP/GFP*^ mice and their wild-type (WT) littermates for FoxP3 together with phalloidin, a marker for polymerized actin, to outline the RPE cells (Fig. 1). At the age of 2 months, WT mice displayed a regular cobblestone pattern of RPE cells with no FoxP3 expression (Fig. 1A, left panel, and 1B at higher magnification). The pattern of the RPE morphology remained stable when analyzed at the age of 8 months in WT mice (Fig. 1A, B, second panel from left) but with patchy FoxP3 expression and localization restricted to the cytosol (Fig. 1B, second panel from left). In 8-month-old *Cx3cr1*^*GFP/GFP*^ mice, which still possess a regular hexagonal pattern of RPE cells, a more homogenous FoxP3 expression was observed, with localization shifted predominantly to the nucleus. (Fig. 1A, B, fourth panels from left showing two different magnifications). At 12 months of age, *Cx3cr1*^*GFP/GFP*^ mice showed enlarged, irregularly shaped RPE cells containing up to three nuclei, with an intense, homogeneous, and exclusively nuclear localization of FoxP3 (Fig. 1A, B, right panels). The structural changes correspond to those reported by Chen et al. [82] and might indicate ongoing repair activities for defects that occurred to the epithelium. The WT mice of the same age showed very mild signs of RPE degeneration, mainly in isolated cells displaying two nuclei (Fig. 1A/B; second panels from the right). While FoxP3 expression in WT mice does not appear to be increased beyond the levels observed at 8 months, at 12 months FoxP3 was exclusively localized in the nuclei. A combined DAPI/FoxP3 staining in *Cx3cr1*^*GFP/WT*^ retinas at the age of 12 months confirmed the localization of FoxP3 to the nucleus (Fig. 1C). Thus, although after 8 months of age, cytosolic FoxP3 expression was induced in the RPE, progressive degeneration of the RPE resulted in FoxP3 translocation to the nucleus.

As FoxP3 is a transcription factor, we proposed that its upregulation and localization to the nucleus promote changes in the expression of pro-inflammatory cytokine genes in the RPE. Therefore, we investigated mRNA expression of FoxP3, IL-1β, MCP-1 and Cxcl1 at 8 and 12 months in RPE/choroid mRNA probes [the time points when FoxP3 expression was enhanced in both, WT and *Cx3cr1*^*GFP/GFP*^ mice (Fig. 1D)]. We compared the expression levels between WT and *Cx3cr1*^*GFP/GFP*^ mice at different time points (Additional file 1: Figure S1) and between the two genotypes at the different time points (Fig. 1D). These data show that at the stage of RPE degeneration and nuclear FoxP3 localization in 12-month-old *Cx3cr1*^*GFP/GFP*^ mice, expression of FoxP3 and the pro-inflammatory factors IL-1β, MCP-1 and CXCL1 (the mouse equivalent to human IL-8; [83, 84] was significantly increased compared to WT mice. Additionally, while the expression of IL-1β, MCP-1 and CXCL1 remained stable over time in WT mice, a significant upregulation was observed in *Cx3cr1*^*GFP/GFP*^ mice between 8 to 12 months of age. The other factors of our panel did not reach significance in the comparison of WT with *Cx3cr1*^*GFP/GFP*^ mice, as the WT mice also showed increased FoxP3 and cytokine expression levels with age. A comparison between the genotypes revealed an increased expression of FoxP3 and all cytokines at the age of 12 months (Additional file 1: Figure S1) in *Cx3cr1*^*GFP/GFP*^ mice compared to wild-type controls.

**Fig. 2:**
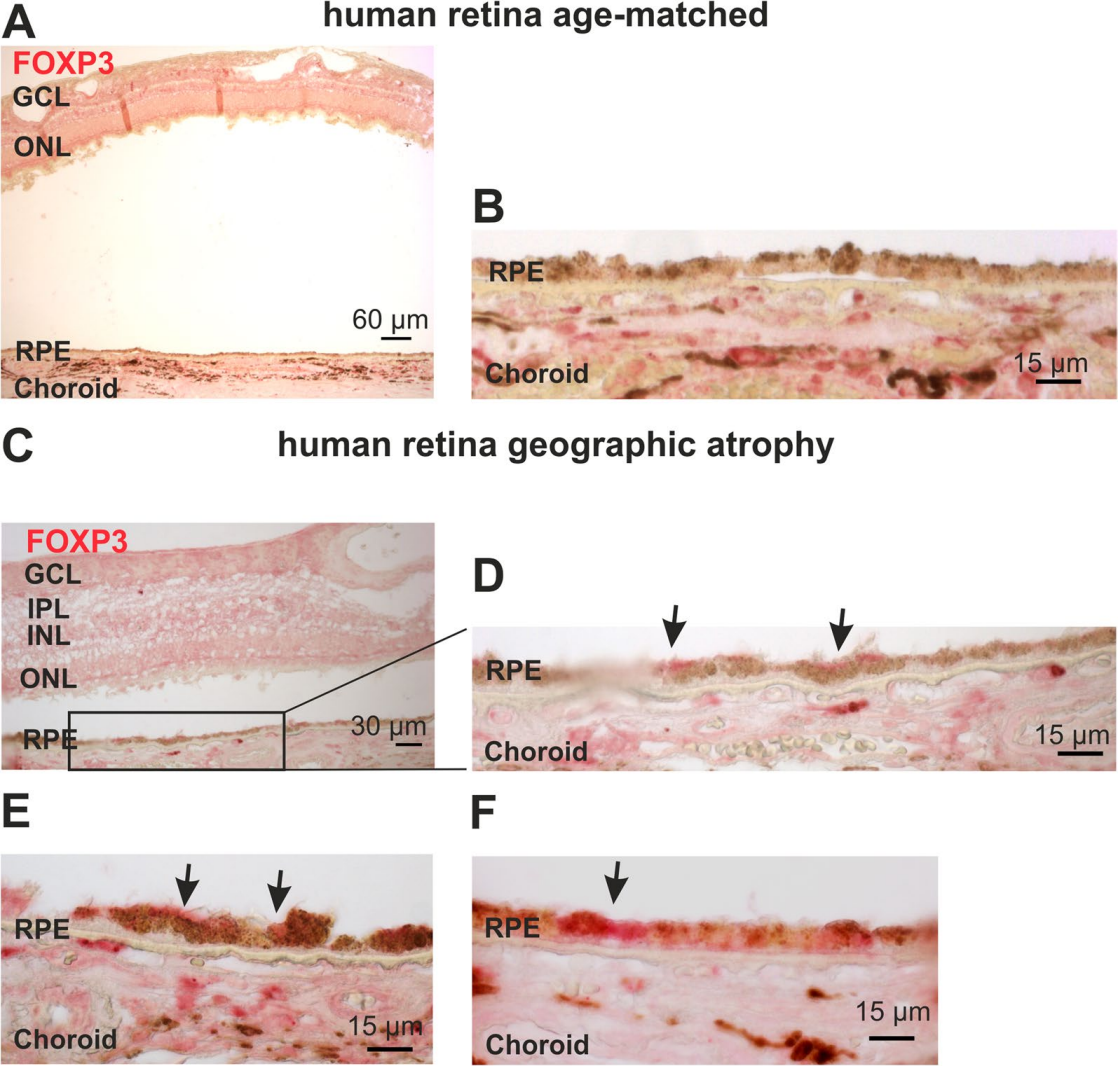
FoxP3 expression in human retinal tissue. **A**, **B** Sagittal sections from age-matched human retinal samples without AMD stained for FoxP3 (red), (**A**) overview of an aged human retina with no retinal degeneration, photoreceptor outer segments are missing (artifact due to the fixation of the eyeball), **B** higher magnification of an area of the same retina as in A. **C**, **D** Sagittal sections from human retina with geographic atrophy stained for FoxP3 (red)**.** Sagittal section from a human retina of a patient with geographic atrophy, at lower magnification (**C**) for an overview (photoreceptor outer segments are missing, artifact due to the fixation of the eyeball), **D** an area at higher magnification from the same section as, arrows indicate RPE cells with FoxP3 expression. FoxP3-positive T cells are also detectable in the blood vessels and represent a positive control for the antibody. **E**, **F** Higher magnifications from sagittal sections of AMD patients comparing areas with RPE atrophy (E; from the same retina as in **D**) and intact RPE layer (**F**). (GCL = ganglion cell layer; IPL = inner plexiform layer; INL = inner nuclear layer; OPL = outer plexiform layer; ONL = outer nuclear layer; RPE = retinal pigment epithelium). N = 3 (negative control with secondary antibody and FastRed staining only; positive control with T cells in a lymph node are shown in Additional file 1: Fig. S2)

Since this mouse model with subretinal inflammation, one of the hallmarks for dry AMD, demonstrated heightened FoxP3 expression and nuclear localization in conjunction with pro-inflammatory cytokine expression, we analyzed retinal sagittal sections from human donors with dry AMD and healthy, age-matched donors using immunohistochemical staining for FoxP3 expression (Fig. 2). In healthy aged human retinas (*n* = 4) we found no FoxP3 expression in the RPE (Fig. 2A, B; examples from four eyes). In contrast, in all human sagittal sections from AMD patients (*n* = 3), FoxP3 was expressed in an area with still intact RPE (Fig. 2D, E). These observations are in conjunction with our findings in the RPE of 8-month-old wild type and *Cx3cr1*^*GFP/GFP*^ mice (Fig. 1A, B). In addition, we found FoxP3 expression in the RPE of AMD donor eyes in an area with partial destruction of the RPE layer (Fig. 2E, F; examples from two eyes), which corresponds to our observations from 12-month-old *Cx3cr1*^*GFP/GFP*^ mice (Fig. 1A, B). Our antibodies and staining methods were verified in a tissue sample from a human lymph node as a positive control, and the staining with the secondary antibody only as a negative control (Additional file 1: Fig. S2).

**Fig. 3:**
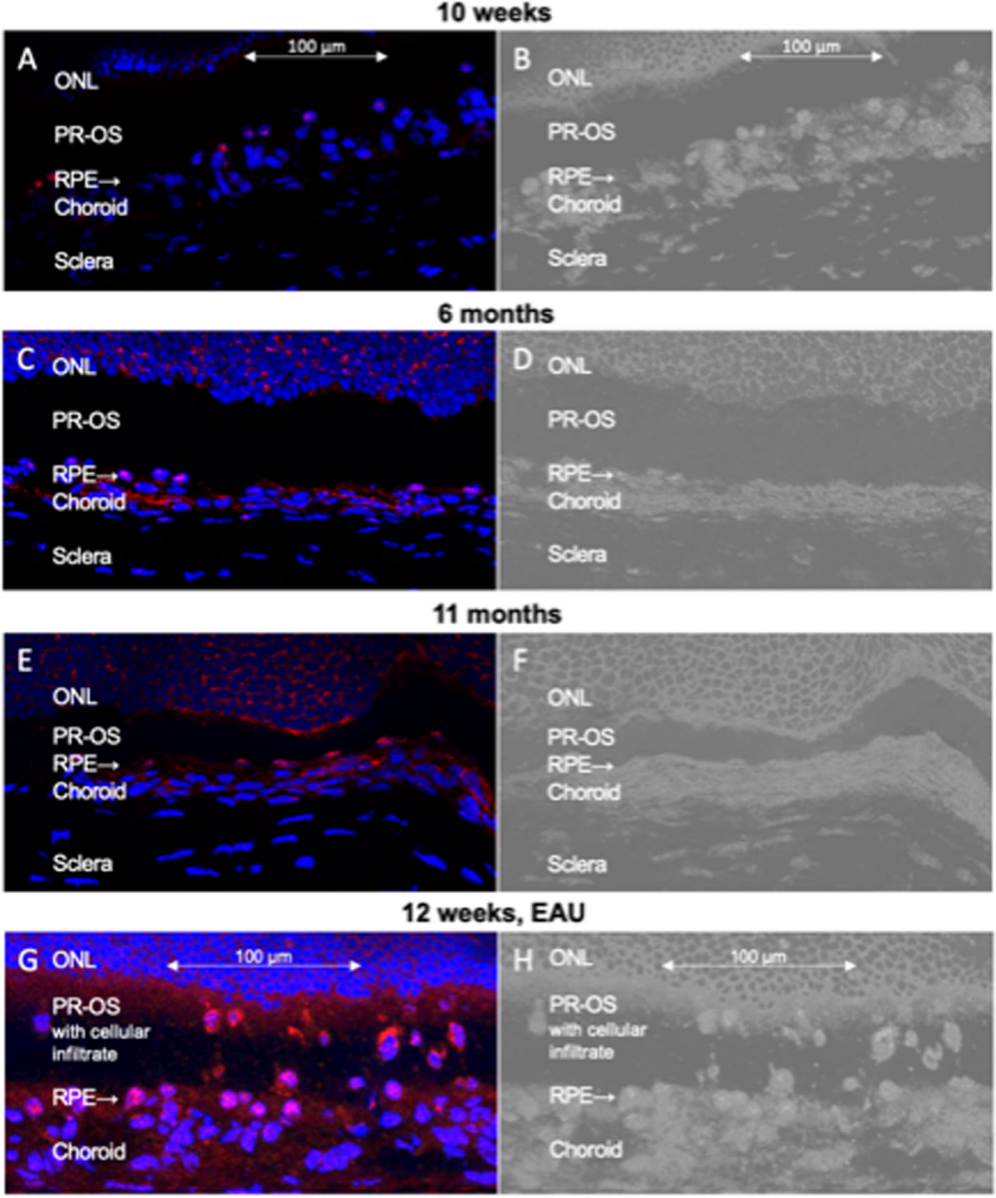
FoxP3 expression in the retina of aged rats or rat eyes with uveitis. **A** FoxP3 (red) expression in 10-week-old rats. Blue = DAPI. **B** Same as A, but darkfield to show the retinal structure. **C** FoxP3 (red) expression in 6-month-old rats. Blue = DAPI. **D** Same as C, but darkfield to show the retinal structure. **E** FoxP3 (red) expression in 11-month-old rats. Blue = DAPI. **F** Same as E, but darkfield to show the retinal structure. **G,** FoxP3 (red) expression in 12-week-old rats after induction of experimental uveitis, with T cells and leukocytes invading the photoreceptor layer from the choroid via the RPE. *N* = 3 animals per age group. **H** Same as G, but darkfield to show the retinal structure. ONL: outer nuclear layer, PR-OS: outer segments of photoreceptors, RPE: retinal pigment epithelium

As the WT mice appeared to show de novo FoxP3-expression followed by translocation into the nucleus with age, we investigated FoxP3-expression in the RPE from retinal sections of albino rats (Lewis rats) between the ages of 10 weeks to 11 months (Fig. 3). Interestingly, in albino rats, FoxP3 expression was already apparent by 10 weeks. The lack of pigment might cause enhanced stress for the RPE of these animals that had developed retinal degeneration in the past when they were not yet housed in transparent shaded cages (our own unpublished observations). In Fig. 3C, E, and G, cells with cytosolic expression of FoxP3 can be detected in the choroid, indicating the presence of effector T lymphocytes. In addition, this model verifies the specificity of the primary antibody used. Figure 3G shows cytosolic and nuclear FoxP3-positive cells invading the layer of photoreceptor outer segments, accompanied by a strong upregulation of FoxP3 in the RPE. In previous studies, these invading cells have been identified as regulatory T cells with nuclear FoxP3 localization and effector T cells with cytosolic FoxP3 [85, 86].

The quantification of FoxP3 expression by RPE cells in rat retinas was determined as % overlay of DAPI and FoxP3 staining of the nuclei (*n* = 15) using ImageJ software [80]. The levels of FoxP3 expression in RPE cells were found similar in 10-week-old (32.4%, *n* = 19) and 6-month-old (28.4%, *n* = 15) rat retinas and significantly increased to the age of 11 months (63.5%, *n* = 6), with *p* = 0.0011 for 10 weeks to 11 months, and *p* < 0.0001 for 6 months to 11 months. Interestingly, in 12-week-old eyes with experimental autoimmune uveitis FoxP3 expression in RPE cells was comparable to 11-month-old untreated rats (62,6%, *n* = 10, Fig. 3G).

**Fig. 4:**
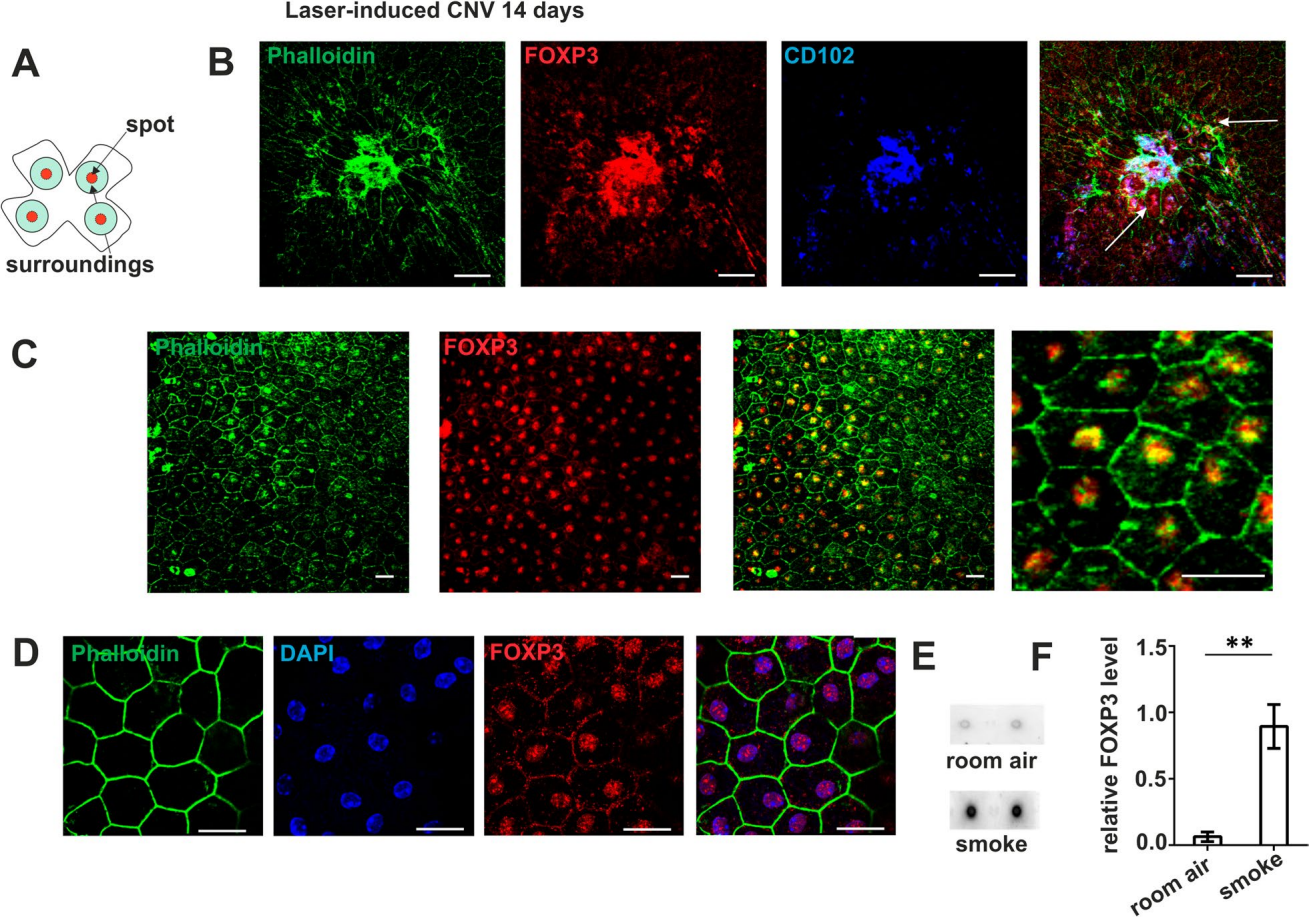
FoxP3 expression in choroidal neovascularization and after long-term smoke exposure in mice. Laser-induced choroidal neovascularization (CNV) in a mouse eye, flatmount preparation of the RPE prepared 14 days after laser burn. **A** Scheme of flatmounted RPE/choroid illustrating the arrangement of laser burns, peri-lesion area (spot) and periphery (surroundings). **B** Immunofluorescence staining (as indicated) of a mouse RPE flatmount with a laser scar showing RPE cells with distorted borders (phalloidin green), FoxP3 (red) positive cells marked with white arrows and blood vessels (CD102 blue). Scale bar represents 50 µm. **C** RPE structure and FoxP3 expression in the peri-lesion area (three left panels) and at higher magnification (right panel); FoxP3 in red and phalloidin in green. Scale bar represents 20 µm. **D** Localization of FoxP3 in the nucleus in the laser CNV model; phalloidin (green), DAPI (blue) and FoxP3 (red) verify the localization of FoxP3 in the nucleus. Scale bar represents 20 µm. **E**,** F** FoxP3 levels under the influence of cigarette smoke in mice. Mice have been exposed to cigarette smoke for 6 months according to Woodell et al*.* [88]. **E** Dot blots for FoxP3 from extracts of RPE/choroid. **F** Quantification of FoxP3 levels. Dot blots of RPE/choroid extracts were probing with anti-mouse FoxP3 antibody, using GAPDH for normalization (**p < 0.01; *N* = 3 per condition)

Another mouse model with relevance for AMD is one with laser burn-induced choroidal neovascularization (CNV) that shows features of wet AMD in humans. It combines a strong inflammatory response with the formation of new blood vessels from the choroid. In contrast to the *Cx3cr1*^*GFP/GFP*^ mouse model, the laser-induced CNV model includes a stronger inflammatory reaction. To induce neovascularization, four laser spots that break the RPE barrier were placed around the optic nerve head (Fig. 4A). Fourteen days later, flatmount preparations of the outer retina were stained with phalloidin (green) to indicate the cell borders, anti-CD102 (blue) to visualize newly developed blood vessels, and anti-FoxP3 (red) (Fig. 4A, B). An overview of the lasered area (Fig. 4B) shows the newly developed blood vessels in white due to the overlay of the three different fluorophores (right), and FoxP3-positive RPE cells (red staining) at the border of the CNV as well as in more peripheral areas. At a greater distance to the laser spot (Fig. 4C), the RPE cells were uniformly FoxP3-positive and, as in the *Cx3cr1*^*GFP/GFP*^ mouse model, FoxP3 was localized to the nuclei (Fig. 4D). We have previously characterized this area as a peri-lesion around the CNV, containing cells that have lost junctional markers and/or their normal hexagonal shape [87]; hence, it was not surprising that FoxP3 expression and nuclear localization was induced in these RPE cells. Thus, in mouse models, we found that both, aging and age-dependent degeneration of RPE cells, as well as laser burn-induced RPE injury followed by neovascularization, induced FoxP3 expression in RPE cells with a major localization in the nucleus.

As a final mouse model of AMD-like pathology, we investigated FoxP3 expression in RPE of mice exposed to constant cigarette smoke. In these mice, we have reported RPE alterations, which include changes in RPE signature gene expression as well as mitochondrial changes indicative of oxidative damage [88]. We found eightfold increased levels of FoxP3 in extracts of RPE/choroid from mice that have been exposed to cigarette smoke ("passive smokers") for 6 months when compared to room air-maintained animals (Fig. 4E, F).

In this manuscript, we also compared confluently grown with non-confluent ARPE-19 cells, assuming that non-confluent RPE cells would represent a more stressed condition. This assumption is based on data published by others, showing that ARPE-19 cells grown as a stable monolayer exhibit gene expression profiles more comparable to those of native primary RPE cells, while the non-confluent cells represent a destabilized condition that leads to rapid changes of the phenotype and loss of RPE differentiation [89, 90].

**Fig. 5:**
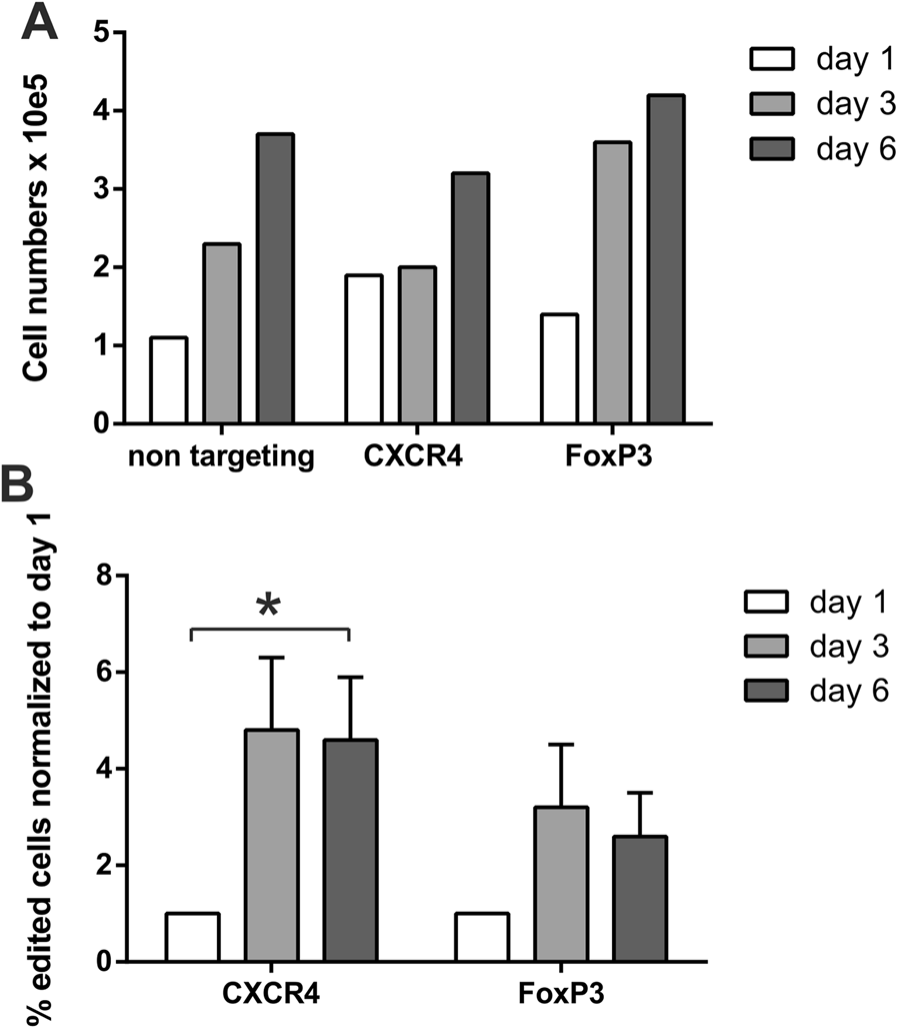
Effect of FoxP3 deletion in ARPE-19 cells using the CRISPR/Cas9 technology. **A** Total cell numbers of ARPE-19 cells after gene editing; cell numbers pooled from all investigated samples over the observation period of 6 days (note cells numbers are to be multiplied with 10^5^). **B** Knockout efficiency calculated from the numbers of edited cells and plotted as change in percentage from day one (means ± SEM; *p < 0.05; *N* = 4)

To investigate the function of FoxP3 expression in ARPE-19 cells, we used CRISPR/Cas9 editing to abolish the FoxP3 gene and, as a control, we knocked out the CXCR4 receptor using the recently established method [91] (Fig. 5). After genome editing, the cells were further cultured and the editing efficiencies as well as the numbers of viable cells were monitored at day 1, 3 and 6 after CRISPR editing and compared to cells electroporated with non-targeting control Cas9 RNPs (Fig. 5A). The total number of viable cells was comparable in non-targeting, CXCR4 KO and FoxP3 KO cell cultures. For comparison, the proliferation rate of untreated ARPE-19 cells over 6 days was determined and is now shown as Additional file 1: Figure S3. Importantly, since Cas9 RNPs remain in the cells for up to 72 h and can introduce further mutations within this time window [92] CRISPR/Cas9 editing of the FoxP3 gene resulted in a peak of 18% edited cells at day 3 and then declined again. Measuring the KO efficiency in percent compared to to day one, there is no significant change in the FoxP3 KO group. In contrast, CXCR4 KO cells reached their peak of editing efficiency at day 3 and remained stable with subsequent significantly increased KO efficiency normalized to day one (Fig. 5B). The reason for the reduced frequency of FoxP3-knockout cells over time could be that the non-edited cells within this condition have a cell growth or survival advantage compared to FoxP3 KO cells, thereby outcompeting the FoxP3 KO cells. Overall, our results suggest that FoxP3 expression is beneficial for cell growth in RPE cells.

**Fig. 6:**
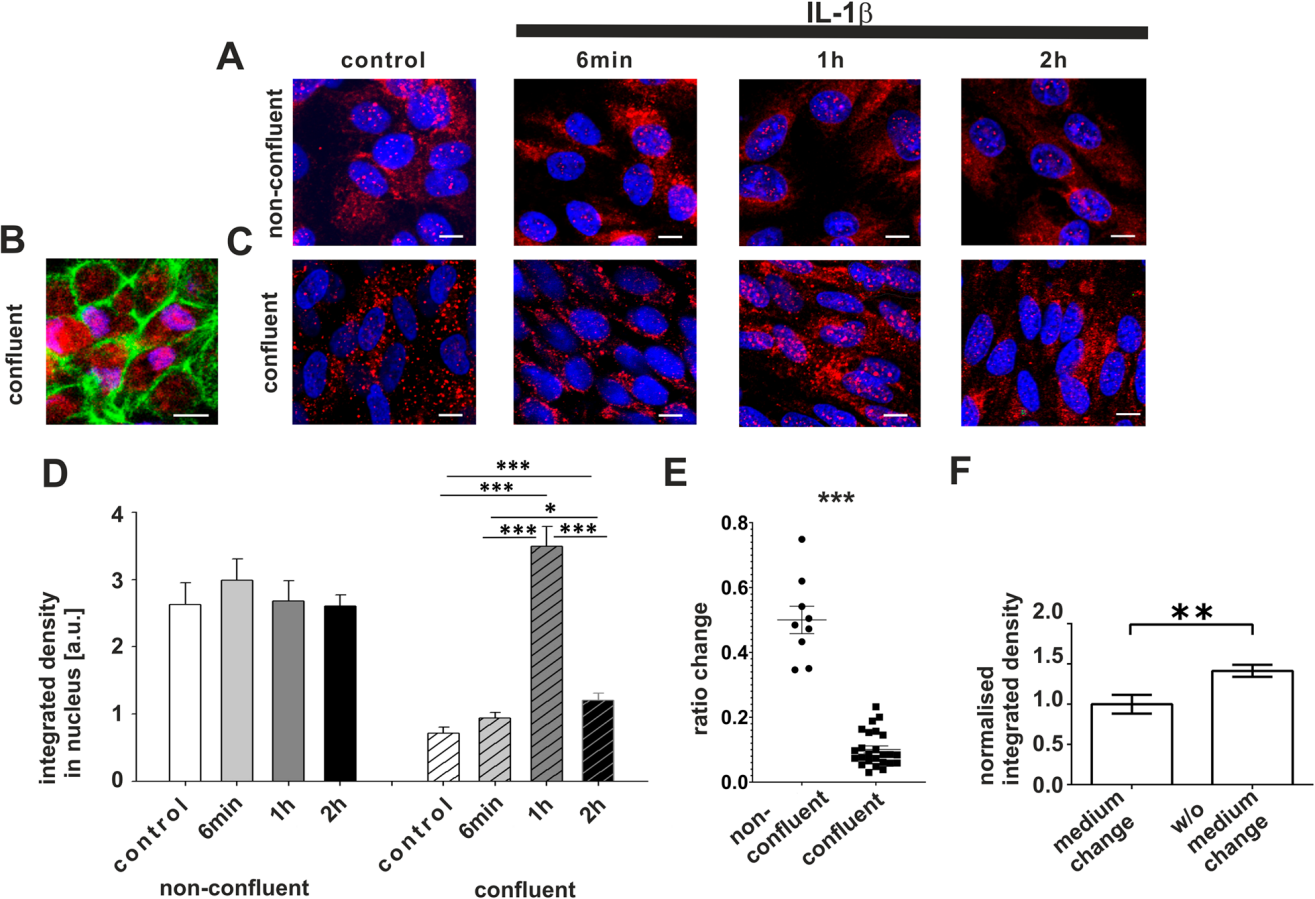
IL-1β treatment of ARPE-19 cells results in FoxP3 translocation from the cytosol to the nucleus. **A** Constitutive FoxP3-expression (red) in non-confluent ARPE-19 cells (blue = DAPI staining of the nucleus) at different time points after IL-1β (100 ng/ml) application: before, 6 min, 1 h and 2 h after IL-1β addition to the culture medium. Scale bar represents 10 µm. **B** Constitutive FoxP3-expression (red) in confluent iPS-RPE cells with a transepithelial resistance of 600 Ωcm^2^ (blue = DAPI staining of the nucleus, green = phalloidin to mark the cell borders). Scale bar represents 10 µm. **C** FoxP3 (red) in confluent ARPE-19 cells (blue = DAPI staining of the nucleus), same treatment as in **A**). Scale bar represents 10 µm. **D** Quantification of FoxP3 in the nucleus by determining the integrated density of the red pixels inside the nucleus (DAPI-stained area) and comparing different time points for non-confluent and confluent ARPE-19 cells. All numbers are given as means + SEM in arbitrary unit (a.u.), Student’s t-test, *p < 0.05; ***p < 0.001, *N* = 21–53). **E** IL-1β-evoked rises in intracellular free Ca^2+^. Ca^2+^-transients were measured using the Ca^2+^-sensitive dye fura-2 at the peak of the Ca^2+^ rise and plotted as changes in the ratio of the fluorescence of the two excitation wavelengths 340 and 380 nm: left group represents the non-confluent and right group the confluent cells (means ± SEM, ***p < 0.001; Mann–Whitney *U* test). **F** comparison of FoxP3 protein in confluent ARPE-19 cells that have been maintained under standard conditions and those that were kept for 14 days without changing the culture medium as stressor (quantification analogous to Fig. 6D by measuring integrated density of red pixels in the nucleus data are normalized to cells with medium change (standard conditions); means + SEM, Student’s *t* test, *p* values **p < 0.01, *N* = 20)

Taken together, thus far, we have shown that FoxP3 expression and localization to the nucleus is induced in RPE cells in mouse models of AMD, as well as in donor retinas from AMD patients, and that FoxP3 is required for growth of cultured ARPE-19 cells. To further investigate the importance of FoxP3 expression for RPE cells, we used cultured RPE cells. Human RPE cells differentiated from inducible stem cells of healthy donors (iPS-RPE), as well as cells of the established ARPE-19 cell line, showed constitutive expression of FoxP3 with cytosolic and/or nuclear localization (Fig. 6A–C). We selected the ARPE-19 cell line as a model system to study the regulation of FoxP3 in response to AMD-like stressors, as we have previously shown that the anaphylatoxins C3a and C5a modulate FoxP3 phosphorylation in these cells [16]. In order to elucidate a possible link between FoxP3 expression/localization and AMD pathology, we investigated the effects of IL-1β, a major macrophage-derived pro-inflammatory cytokine. A recent hypothesis postulates that a chronic, local presence of monocytes compromises the immune regulatory capacity of the RPE, potentially shifting the phenotype of the RPE from a regulatory type to an immune stimulating effector type [12]. IL-1β is one of the monokines that might lead to the loss of the immune regulatory phenotype of the RPE and has been detected in many AMD-relevant models [38, 50, 93, 94]. Thus, we treated confluent and non-confluent ARPE-19 cells, which, respectively, simulate the stages of stable and destabilized RPE, with IL-1β and investigated its impact on FoxP3 expression/localization and subsequent cytokine/chemokine secretion.

To evaluate the shift of constitutively expressed FoxP3 from the cytosol to the nucleus in response to IL-1β, we analyzed the percentage of FoxP3 in the nucleus of ARPE-19 cells. For that purpose, confluent and non-confluent cells were exposed to IL-1β (100 ng/ml), and the localization of FoxP3 was investigated by immunofluorescence staining and counting pixels of FoxP3 staining (Fig. 6D). Even under untreated control conditions, confluent and non-confluent cells showed differences in FoxP3 localization (Fig. 6A, C). While in both confluent and non-confluent culture conditions, a significant amount of FoxP3 expression was observed, confluent cells showed predominant FoxP3 localization in the cytosol with a minor fraction in the nuclei (Figs. 6C, 10A). In contrast, non-confluent cells had a larger proportion of FoxP3 in the nuclei (Figs. 6G, 10A). Stimulation with IL-1β also had different effects on cells in these two stages. In non-confluent cells, FoxP3 localization in the nuclei remained unchanged after IL-1β application (Fig. 6A, D). In contrast, in confluent cells IL-1β application led to a transient increase in nuclear localization of FoxP3 that returned to baseline levels after 2 h, even though IL-1β was still present in the culture media (Fig. 6C, D). Thus, confluent cells seem to represent a baseline state that can become activated after a 1 h exposure to IL-1β, whereas stressed, non-confluent cells already appear to be maximally activated. To rule out the possibility that non-confluent cells might fail to express IL-1β receptors and are thus unable to respond to IL-1β, we measured changes in intracellular free Ca^2+^ as a second messenger by fluorescence microscopy-based cell imaging with the Ca^2+^-sensitive fluorescence dye fura-2 as the probe. Interestingly, while all non-confluent cells responded to stimulation with IL-1β with a strong increase in intracellular free Ca^2+^, only a very small proportion of confluently grown cells were IL-1β-reactive and showed significantly smaller Ca^2+^ peaks (Fig. 6E). Taken together, this set of experiments showed that IL-1β induced a transient shift of FoxP3 from the cytosol into the nucleus in confluent RPE cells, whereas the non-confluent cells that displayed a permanent high FoxP3-expression in the nucleus did not experience increased nuclear FoxP3 expression, even though responded with a strong Ca^2+^ signal to only 40 s contact with IL-1β. As another stressor, we kept monolayers of ARPE-19 cells for 14 days without changing the FCS-containing medium**.** These confluent cells without medium exchange also had increased FoxP3 expression in the nucleus after 14 days (Fig. 6F).

**Fig. 7:**
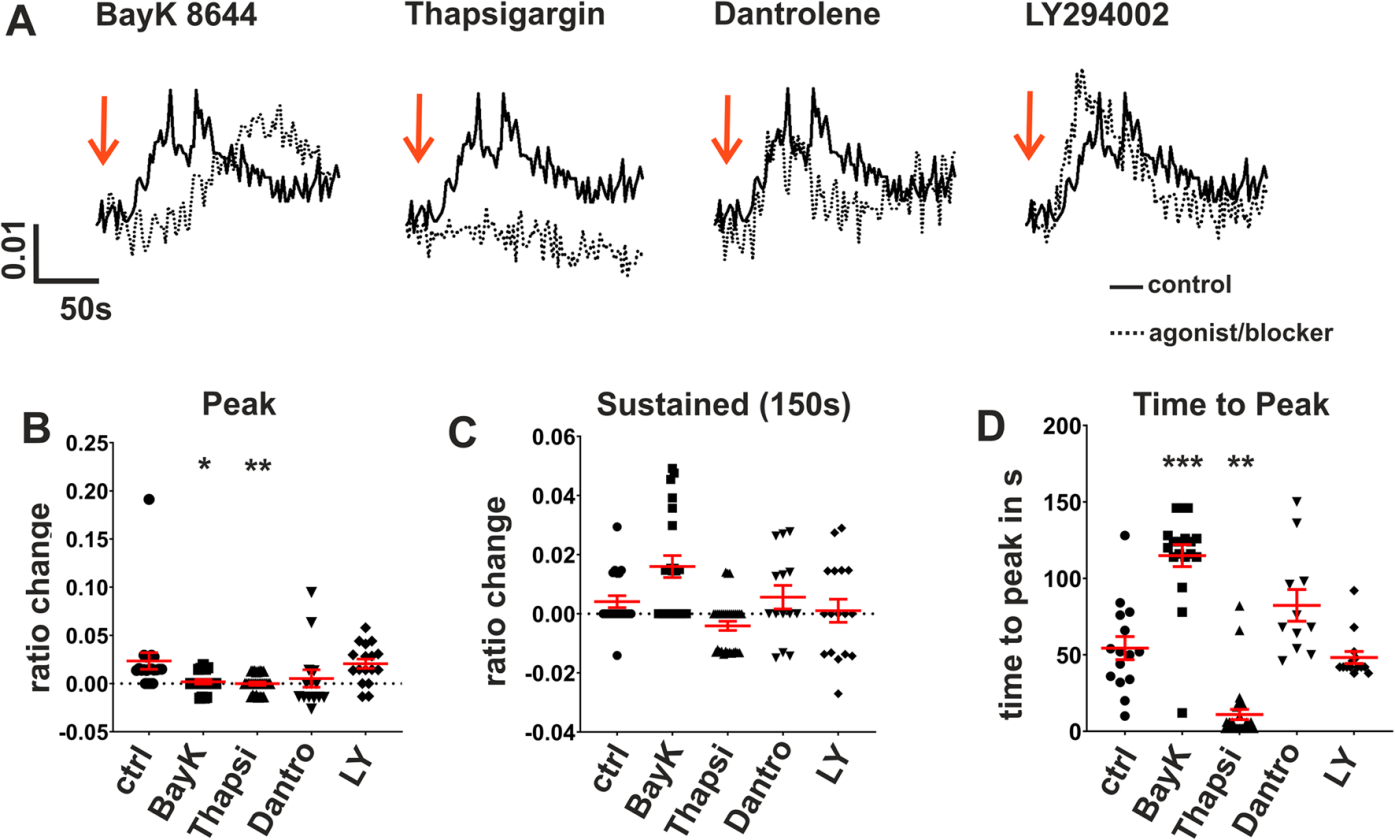
Analysis of IL-1β-evoked Ca^2+^-transients in non-confluent ARPE-19 cells. Intracellular free Ca^2+^ was measured in non-confluent ARPE-19 cells using the Ca^2+^-sensitive fluorescence dye Fura-2; changes in intracellular Ca^2+^ were plotted as changes in the fluorescence ratio of the excitation wavelengths. **A** Representative raw data from single-cell experiments (solid line represents IL-1β (100 ng/ml) alone; dotted lines IL-1β after 5 min preincubation with the following inhibitors of Ca^2+^-signaling: 10 µM BayK 8644 (blocker of L-type Ca^2+^ channels), 1 µM thapsigargin (blocker of sarcoplasmic Ca^2+^-ATPase to empty intracellular Ca^2+^-stores); 1 µM dantrolene (blocker of ryanodine receptors); 50 µM Ly294002 (blocker of PI3-kinase). **B** Comparison of IL-1β-evoked Ca^2+^ sustained elevation. **C** Comparison of IL-1β-evoked Ca^2+^-peaks. **D** Comparison of time-to-peak of IL-1β-evoked Ca^2+^ transients. (BayK = BayK8644; Thapsi = thapsigargin; Dantro = dantrolene; LY = Ly294002). Data are presented as mean values ± SEM. Statistical significance was calculated using Mann–Whitney *U* test

Considering that the non-confluent cells might reflect the stage at which RPE cell loss occurs in AMD [95], we hypothesized that IL-1β-dependent increase of intracellular free Ca^2+^ as a second messenger is essential for RPE cells that are more close to die. Thus, we investigated the underlying signaling mechanisms in detail. We studied Ca^2+^ signaling in non-confluent ARPE-19 cells using blockers for ion channels and intracellular Ca^2+^ stores to investigate whether RPE cells with a predominant nuclear FoxP3 expression display the specialized molecular mechanisms needed of signal transduction. IL-1β-induced Ca^2+^ increase was dependent on the activation of L-type Ca^2+^ channels through release of Ca^2+^ from cytosolic Ca^2+^ stores and coupling via ryanodine receptors (Fig. 7A–D). The peak of the IL-1β-evoked Ca^2+^-rise was significantly reduced by either the L-type channel blocker BayK8644 (10 µM), after preincubation with the blocker of sarcoplasmic Ca^2+^-ATPase (SERCA) thapsigargin (1 µM) or with dantrolene (1 µM), the blocker of ryanodine receptors. The latter one couples release of Ca^2+^ from cytoplasmic Ca^2+^-stores with activation of L-type channels. L-type Ca^2+^ channel blockade with BayK8644 or ryanodine receptor blockade by dantrolene also slowed down the time-to-peak, indicating that L-Type channels coupling with ryanodine receptors are responsible for the raising phase of the Ca^2+^ signal. The blocker of PI3-kinase Ly294002 (50 µM) had no effect. In summary, both confluent and non-confluent ARPE-19 cells respond to IL-1β but do so in different ways. IL-1β receptor activation leads to nuclear translocation of FoxP3 in confluent cells but to a Ca^2+^ shift in non-confluent cells.

**Fig. 8:**
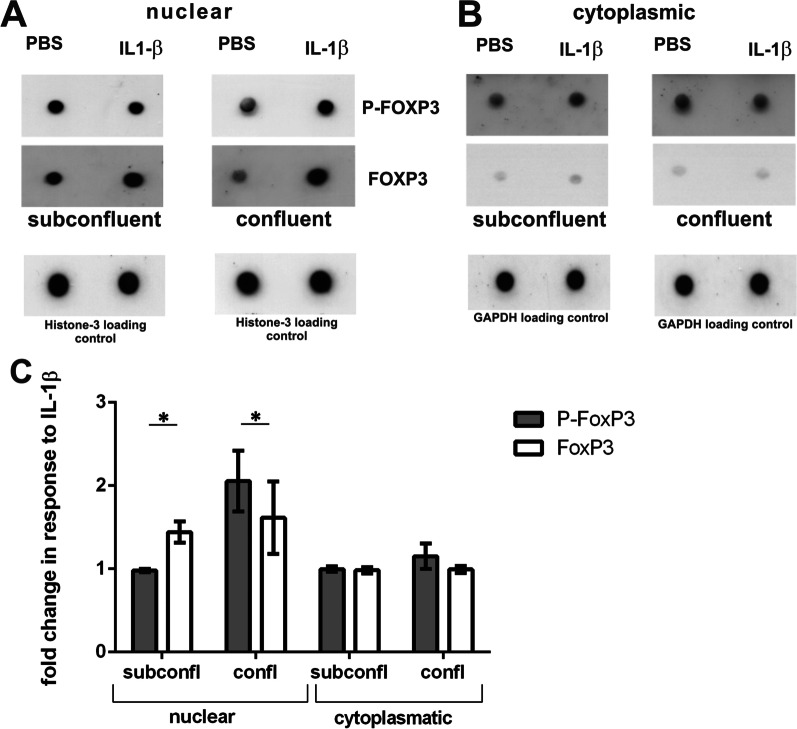
Effect of IL-1β on FoxP3 phosphorylation. **A** Dot blots showing P-FoxP3 in the nucleus of confluent and non-confluent ARPE-19 cells treated for one hour with IL-1β or PBS as control. **B** Dot blots showing P-FoxP3 in the cytoplasm of confluent and non-confluent cells after 1 h IL-1β- or PBS-treatment. **C** Comparison of phosphorylation under different conditions in the two cellular compartments nucleus and cytoplasm, determined by densitometry and normalized to the respective loading controls (histone H3 and GAPDH) (means + SEM, Student’s *t* test, *p* values *p < 0.05)

FoxP3 localization in cells is one simple marker for its activity and has already been correlated with substantial changes in the functional phenotype in both this study and others. Another marker of its activity is the phosphorylation status of FoxP3, that we have previously described in the context of anaphylatoxin-dependent regulation of secretion in RPE cells [16]. Therefore, we measured the proportion of phosphorylated FoxP3 over total FoxP3 from the cytosol and the nuclei and compared these values between confluent and non-confluent cells stimulated with IL-1β. Protein levels were measured in dot blots stained for FoxP3 and phosphorylated (P-)FoxP3, normalized to GAPDH (cytosolic fraction) or Histone 3 (nuclear fraction) (Fig. 8A, B). Using densitometry, we estimated the protein content of P-FoxP3 and total FoxP3 and calculated the ratios (Fig. 8C). In confluent, untreated ARPE-19 cells the amount of P-FoxP3 in the nucleus was higher than in the cytosol. Moreover, confluent ARPE-19 cells showed a higher proportion of P-FoxP3 in the nucleus compared to non-confluent cells (Fig. 8C), even though the confluent cells display less overall FoxP3 localization in the nucleus (Fig. 6). After application of IL-1β, we found a slight decrease of P-FoxP3 in the nuclear fraction of confluent cells, whereas the amount in the cytosol remained unchanged. Again, the non-confluent cells behaved differently. In untreated cells, the proportion of P-FoxP3 in the nucleus was equal to that in the cytosol (Fig. 8C). However, following stimulation with IL-1β, we found a significant increase in the proportion of P-FoxP3 in the nucleus (Fig. 8C), although the amount of total FoxP3 remained unchanged (Fig. 6).

**Fig. 9:**
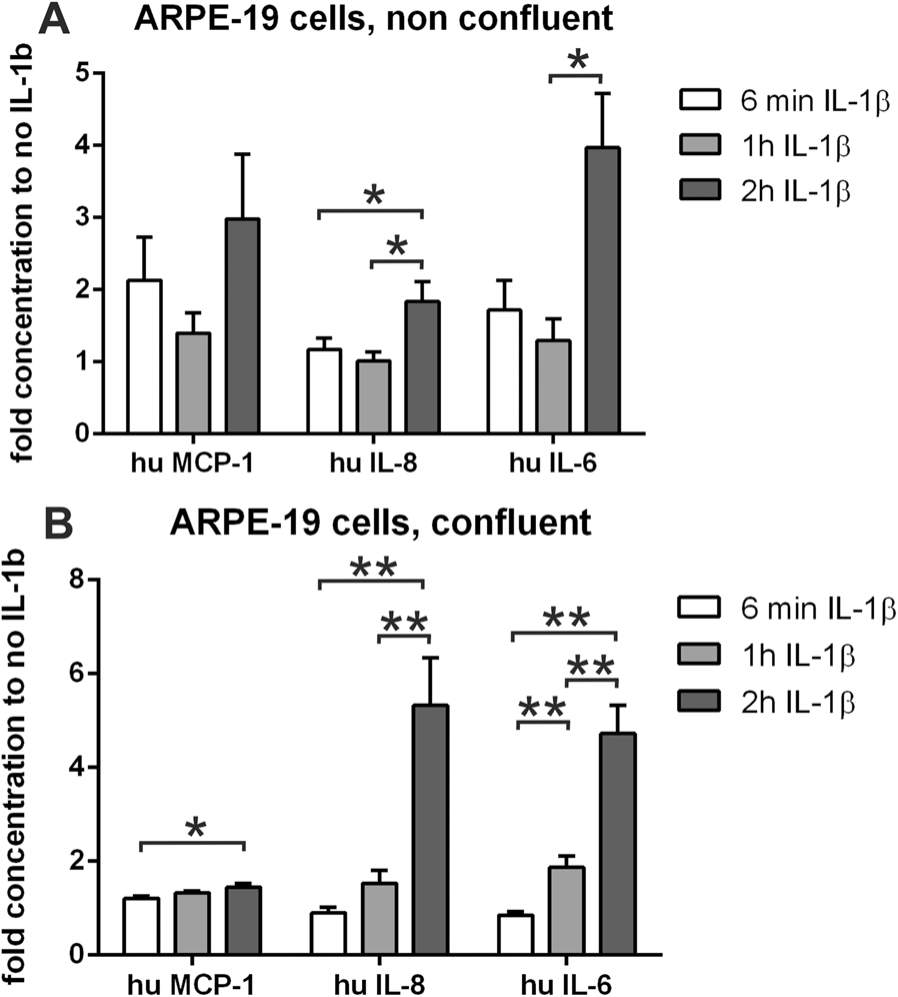
Secretion of cytokines and chemokines by ARPE-19 cells after stimulation with IL-1β. Secretion of MCP-1, IL-8 and IL-6 (pg/ml) by ARPE-19 cells stimulated with IL-1β for 6 min, 1 and 2 h. **A** Non-confluent cells. **B** Confluent cells (IL-6 = interleukin-6; IL-8 = interleukin-8; MCP-1 = monocyte chemoattractant protein-1/CCL2). Triplicate cultures of ARPE-19 were treated as described; culture supernatants were collected at the end points of IL-1β-treatment. Supernatants were pooled and tested for cytokine and chemokine secretion as duplicates in a multiplex bead assay. The final values of duplicate supernatants are calculated by the standard curves of the assay from the median fluorescence intensity of at least 50 beads measured for each analyte and sample and normalized to the cell number of confluent cultures (cytokine concentration of non-confluent cultures × 1.5). The data show the x-fold concentrations (means + SEM) of cytokines compared to the unstimulated cultures (*p < 0.05; **p < 0.01; *N* = 4–6)

To assess whether the corresponding cellular response might contribute to an AMD-relevant RPE cell behavior, we investigated the secretory phenotype of ARPE-19 cells under conditions for which we can correlate the potential impact of FoxP3 by its localization in the nucleus or cytosol. Again, we used confluent and non-confluent cells that were stimulated with IL-1β, and the supernatants were collected at the end of the incubation time and profiles of secreted factors generated (Fig. 9). In general, both confluent and non-confluent showed comparable spectra of secreted factors that were changed by IL-1β stimulation over time (Fig. 9A, B). However, the confluent cells produced much higher amounts (2.5 times up to 20 times higher) of the identified factors at baseline and at the early time points (6 min and 1 h) compared to the non-confluent cells, while the maximal secretion after 2 h of IL-1β-stimulation was similar. Under both culture conditions, IL-1β led to a selected increase in the secretion of IL-8 and IL-6 among the 14 investigated cytokines and chemokines measured as increasing concentrations in the culture medium. However, MCP-1 was only enhanced in non-confluent cells.

**Fig. 10:**
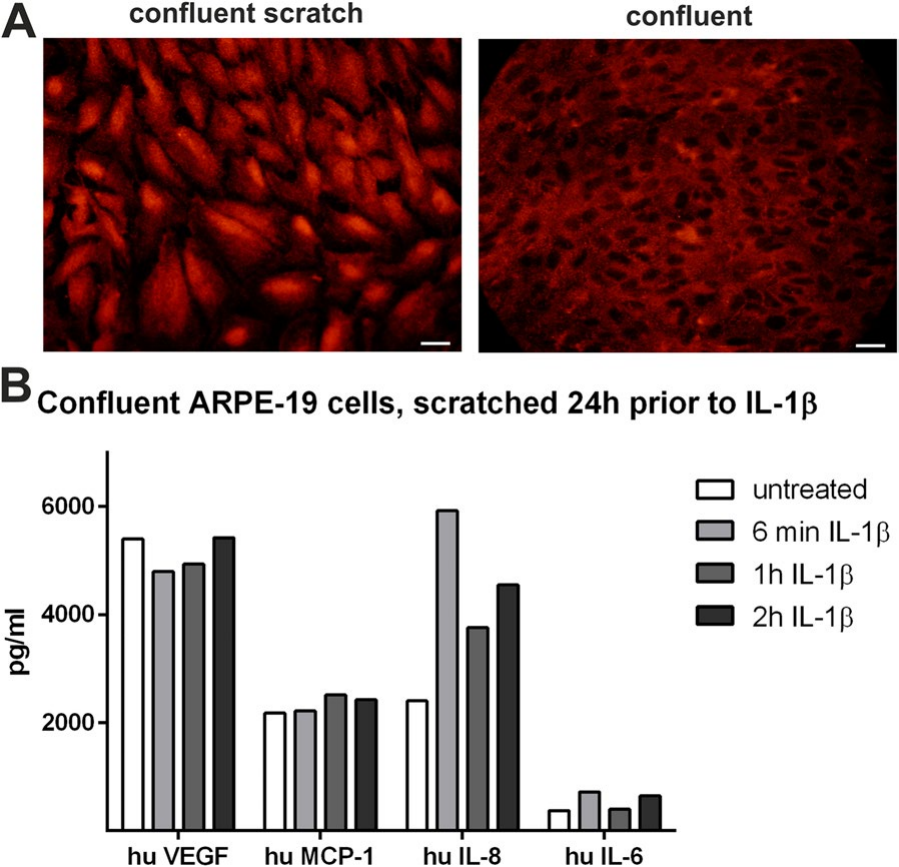
ARPE-19 cells under severe stress conditions like the traumatic destruction of the cell monolayer by a scratch 24 h prior staining for FoxP3. **A** The cell layer of confluently grown ARPE-19 cells was scratched (here: not treated with IL-1β) 24 h prior to staining for FoxP3. The scratched cells showed a translocation of FoxP3 from the cytoplasm to the nucleus (left panel: FoxP3 predominantly localized perinuclear and nuclear), while in confluent, unstressed cells FoxP3 expression is seen in the cytoplasm (right panel: confluent, untreated). Scale bar represents 20 µm. **B** Secretion of VEGF-A, MCP-1, IL-8 and IL-6 (pg/ml) by confluent ARPE-19 where the cell monolayer was scratched 24 h prior to the additional stimulation with IL-1β for 6 min, 1 and 2 h. (IL-6 = interleukin-6; IL-8 = interleukin-8; MCP-1 = monocyte chemoattractant protein-1 (CCL2); VEGF-A = vascular endothelial growth factor-A). Triplicate cultures of ARPE-19 were treated as described; culture supernatants were collected at the end points of IL-1β-treatment. Supernatants were pooled and tested for cytokine and chemokine secretion as duplicates in a multiplex bead assay (*N* = 2). The final values of duplicate supernatants are calculated by the standard curves of the assay from the median fluorescence intensity of at least 50 beads measured for each analyte and sample

In addition to the IL-1β-dependent changes in FoxP3 localization and secretion profiles, we also observed IL-1β-independent stimulation of FoxP3 activity by translocation from cytoplasm to nucleus. After a scratch through the confluent monolayer of ARPE-19 cells, we found a translocation of FoxP3 into the nucleus in cells of the injured culture 24 h later, as well as a secretion profile that compares to the one induced by IL-1β stimulation. In contrast to IL-1β-stimulation, the scratch disrupting the cell monolayer induced VEGF-A secretion in addition to MCP-1, IL-8 and IL-6 in ARPE-19 cells (Fig. 10A, B). Although the cells had been in culture without change of their medium (containing 5% FCS and stable glutamine) for 2 weeks, they secreted high amounts of cytokines and responded differentially to IL-1β-stimulation and scratch. The cytokine concentrations in culture medium were comparable to those from APRE-19 cell cultures only challenged with IL-1β (Fig. 9), while the cytokine expression pattern was changed.

In summary, we showed that FoxP3 activity is differentially regulated under varying conditions that mimic cellular stress of the RPE and local inflammation in AMD (Figs. 9, 10).

## Conclusions

Here we have shown that FoxP3 is expressed in the aged retina and under circumstances of retinal degeneration as well as in acute and chronic inflammation in various species including humans. FoxP3 seems to be an important transcription factor of RPE cells under stress conditions. It is under regulatory control, in part, by oxidative stress, loss of cell–cell contact, or the pro-inflammatory cytokine IL-1β. Oxidative stress (cigarette smoke) and IL-1β can affect the translocation of FoxP3 between the cytosol and the nucleus, as well as its phosphorylation status. As IL-1β changed the FoxP3 activation status, resulting in changed secretory profiles of RPE cells, we conclude that changed secretions (namely MCP-1 and IL-8) are important target genes of that transcription factor. A correlation between cellular confluence and non-confluence in in vivo and in vitro models indicated that cells in intact monolayers, such as those in pre-disease states, or cells even localized at a far distance to areas of degeneration, express FoxP3. However, this expression is mostly localized to the cytoplasm, with weak abundance in the nucleus. In contrast, cells in diseased areas, which possess disrupted monolayers, showed uniformly high localization of FoxP3 to the nucleus. Our data suggest that these differences in the cellular distribution of FoxP3 influence the reactivity of RPE cells to the pro-inflammatory cytokine IL-1β and might represent a basis for the immunologic switch in RPE cells from healthy to a diseased state. Our data further suggested that in cells with abundant nuclear FoxP3, FoxP3-mediated gene expression might be regulated by Ca^2+^-dependent phosphorylation of FoxP3. In cells with a major proportion of FoxP3 present in the cytosol, regulation of FoxP3-mediated gene expression primarily depended on the translocation from cytosol to the nucleus.

We recently reported that FoxP3 protein is not detected in the RPE of young animals. Here we report that with aging, murine RPE cells started to express FoxP3, which was predominantly localized in the cytosol. However, in disease conditions such as experimental uveitis or laser-induced CNV, FoxP3 abundantly localized to the nucleus, which is suggestive of an increased activity of the transcription factor on gene expression in the RPE. Moreover, in the laser CNV model, we documented abundant nuclear FoxP3 in the neovascular areas or zones with loss of RPE cells as well as neighboring RPE peri-lesion areas. Importantly, we also confirmed FoxP3-expression in human RPE from geographic atrophy lesions, which was not detected in age-matched healthy retinas. The time between FoxP3 expression in the RPE and onset of clinical signs of geographic atrophy is currently not known in humans, but is expected to be individually variable due to the differences in lifestyle and genetic background, which could explain the lack of FoxP3 in aged, but healthy human eyes. Thus, FoxP3 appears as a pivotal transcription factor for the RPE associated with aging and conditions of stress. This is further reflected by our investigations of the ARPE-19 cell line, in which we report constitutive FoxP3 expression. We speculate that under culture conditions this human RPE cell line does not reach its final differentiation state that would permit a shut-down of FoxP3 expression, but instead remains at a certain level of permanent cell stress (i.e., similar to the aged RPE in rodents). The observation that the localization of FoxP3 varied with the status of the cell culture supports this hypothesis. In non-confluent (i.e., more stressed) cells, FoxP3 localized predominantly to the nucleus, whereas in cells forming an intact monolayer (i.e., healthy) for more than 2 weeks the majority of FoxP3 was found in the cytosol. ARPE-19 cells can regain a phenotype and transcriptome like native RPE after culture in confluence for months, while we maintained the cells as confluent cultures for up to 3 weeks only [89, 90]. Thus, the not yet terminally differentiated cells may still require FoxP3 expression to retain cellular integrity. The deletion experiments targeting the FoxP3 gene in ARPE-19 support the importance of FoxP3 expression for the resistance of these cells to the stress of CRISPR/Cas9 gene editing by electroporation. Since ARPE-19 cells are of male origin they are hemizygous for the FoxP3 gene encoded on the X-chromosome, resulting in a complete knockout of FoxP3 by CRISPR/Cas9-gene editing in an efficiently targeted cell and reduced recovery of FoxP3-deficient cells. Based on these observations, we propose that FoxP3 expression and its translocation to the nucleus in situations of enhanced stress is essential for the resistance against age-dependent changes or RPE damage, or acute or chronic inflammation like in uveitis.

Co-culturing of RPE cells and lymphocytes results in the induction of regulatory, FoxP3-expressing T cells that support the maintenance of the ocular immune privilege, which requires TGF-β-production by the RPE and helps for the induction of peripheral Tregs [96, 97]. Since TGF-β plays major roles in the immune privilege of the eye and is also produced by RPE cells, FoxP3 expression in the RPE might be induced by autocrine activation with TGF-β [98]. FoxP3 expression of the RPE cells themselves was not yet known when these RPE-lymphocyte co-culture experiments were originally performed. Interestingly, in human and rat T cells, FoxP3 is transiently localized to the cytoplasm of activated effector T cells, and only when they have differentiated to regulatory cells is FoxP3 is found in the nucleus [63, 99–103].

The shift to nuclear localization of FoxP3 in RPE cells under stress conditions might reflect the gain of a regulatory, immunosuppressive phenotype of the RPE, necessary to maintain the ocular immune privilege once the outer blood–retina barrier is endangered. We do not yet know whether we can immediately transfer the knowledge of FoxP3 in regulatory T lymphocytes to the RPE, but so far, our findings support this hypothesis. In Tregs, phosphorylation of FoxP3 at Ser-418 supports their regulatory/suppressive function. Here we have shown that in RPE cells the nuclear FoxP3 is also phosphorylated at Ser-418, likely indicating a highly immune-suppressive RPE-phenotype [104, 105]. In general, we observed a shift of intracellular FoxP3 localization upon IL-1β stimulation. On one hand, we found an increase of FoxP3 in the nucleus in confluent cultures that seemed to be accompanied by a decrease of nuclear phosphorylated FoxP3. On the other hand, in subconfluent cultures we observed no changes of cellular FoxP3 localization but an increase of phosphorylated FoxP3 in the nucleus.

Here, these differences might be related to the different cell culture conditions of ARPE-19 cells grown in transwells (confluent) versus glass cover slips (non-confluent). However, it is worthwhile to mention that under both conditions we see a robust expression of FoxP3. The analysis of the dot blots from transwell-grown cells showed a significant amount of phosphorylated FoxP3 under baseline conditions already. Thus, the strongly increased FoxP3 expression goes along with a large proportion of already phosphorylated FoxP3.

To imitate stress, we further investigated factors that lead to FoxP3 activation and the resulting changes in the secretory activity of RPE cells. Since the RPE forms the border between the immune system and the inner eye as an "educational barrier", RPE cells need to communicate with the cells of the immune system using a common set of cytokines and receptors [4, 49]. Activated macrophages release IL-1β and are thought to be drivers of chronic inflammation leading to AMD [12, 27, 38]; and although expression of the IL-1β receptor on RPE cells was postulated, it was never proven at a functional level [106, 107].

IL-1β-mediated signaling in the RPE was examined in confluent and non-confluent ARPE-19 cells by investigating the intracellular Ca^2+^ increase along with FoxP3 translocation to the nucleus. Confluent cells reacted to IL-1β stimulation with very small Ca^2+^ peaks in only a small subset of cells. In contrast, in response to IL-1β stimulation, non-confluent cells showed a widespread increase in free cytosolic Ca^2+^ as a second messenger that was driven by the release of Ca^2+^ from cytosolic Ca^2+^ stores as well as ryanodine receptor-dependent activation of L-type Ca^2+^ channels [108]. When analyzing FoxP3 localization, confluent ARPE-19 displayed a transient increase of FoxP3 translocation to the nucleus, whereas non-confluent cells with already "constitutive" nuclear FoxP3 did not respond with altered FoxP3 localization. Phosphorylation of FoxP3 at Ser-418 was found to be increased in non-confluent cells and IL-1β stimulation resulted in an increase of phosphorylated nuclear FoxP3, whereas confluent cells already showed maximal levels of nuclear P-FoxP3 that could not be further elevated although the total Foxp3 is lower than in non-confluent cells. Finally, we investigated the effect of risk factors of AMD that induce oxidative stress, like cigarette smoke, on FoxP3 expression/localization in animal models. Mice exposed to intermittent cigarette smoke for 6 months [88], exhibit changes in gene expression in the RPE as well as mitochondrial alteration, but no apparent cell death [88]. In smoke exposed mice, levels of FoxP3 in the RPE were significantly elevated when compared to room air-maintained mice. In an in vitro assay with scratched confluent cell monolayers of ARPE-19 cells, FoxP3 localization was predominantly in the nucleus compared to the non-injured, confluent monolayer cultures. These collective data indicate that damage of the RPE cell monolayer leads to a phenotype of FoxP3 expression that is resembling non-confluent cells. Furthermore, culturing ARPE-19 cells for 14 days without medium change increased the FoxP3 expression, however, with expression in the cytosol. Thus, this pattern of stress-induced FoxP3 activation indicates that RPE cells might need FoxP3 expression in situations of oxidative stress, inflammation and trauma for their function and probably also for their survival.

We hypothesize that these events are also represented by the animal models with AMD relevance, where we could identify FoxP3 expression and translocation into the nucleus. The aged retina with increased oxidative stress and the *Cx3cr1*^*GFP/GFP*^ mouse are both models with a decreased innate immune regulation and function, which leads to a loss of RPE cells with increasing age. The laser-induced choroidal neovascularization model is driven by a strong local inflammatory response caused by cell necrosis and the break of the outer blood–retina barrier. Moreover, retinal laser burn causes a breakdown of the ocular immune privilege even in the non-treated partner eye [109]. In each of these models, the stressor leads to loss in RPE integrity, which is accompanied by translocation of FoxP3 into the nucleus.

To answer the question of whether FoxP3-dependent regulation of gene expression is required for the RPE to control local immune responses, we investigated changes in the secretory profile of ARPE-19 cells under conditions correlated with FoxP3 activation. First, we found that confluent APRE-19 cells showed much stronger reactions of secretory activity compared to non-confluent cells, which is suggestive of a more stable phenotype when cells have reached confluence. Under stimulation with IL-1β, both confluent and non-confluent cells increased the secretion of IL-6 and IL-8, while non-confluent cells only showed increased MCP-1 after 2 h of IL-1β exposure. In physiologically relevant conditions, the secretion of MCP-1 by already damaged/stressed RPE cells in response to IL-1β released from macrophages could in turn further attract and stimulate monocytes and thus create a feedback loop perpetuating inflammation. The changes of mRNA expression in RPE/choroid samples with age in *Cx3cr1*^*GFP/GFP*^ mice support this conclusion. Here, increased levels of FoxP3 coincide with increased levels of IL-1β. Indeed, Sennlaub et al. [15] had previously demonstrated that increased levels of MCP-1 lead to increased numbers of monocytes in the outer retina. Increased numbers of monocytes, along with increased IL-1β expression, might in turn enhance Cxcl1 expression, the mouse analogue of human IL-8, in concordance with our data shown here. Interestingly, when compared with wild-type mice, the Cxcl1 expression is lower in the *Cx3cr1*^*GFP/GFP*^ mice at 8 months of age. We interpret this effect as an early onset of interaction of the RPE with immune cells in the *Cx3cr1*^*GFP/GFP*^ mouse model, where both, monocytes and T cells, are dysregulated by the gene deficiency.

Another way to stress ARPE-19 cells is the destruction of the confluent monolayer of cells by a scratch, which disrupts the contact between the cells, creating an injury comparable to laser burn in animal models. The damage induced the secretion of high levels of MCP-1, IL-8, IL-6 and especially VEGF-A that could not be further increased by addition of IL-1β. VEGF-A was only induced by the scratch-mediated cell destruction, and not solely by IL-1β application. The expression of this panel of genes also occurred in the retina of the *Cx3cr1*^*GFP/GFP*^ mouse model with relevance for geographic atrophy. In the degenerative state of the retina, the cytokines MCP-1, IL-8 (the mouse homologue is Cxcl1), and IL-1β, were significantly increased. As IL-8 is known to play a role in autocrine self-protection of the RPE against degenerative impacts, the FoxP3-driven reaction likely represents a rescue mechanism [49, 110].

The secretion profile indicates a pro-inflammatory phenotype of the RPE when FoxP3 is active. As IL-1β is a driver towards that phenotype and is secreted by monocytes that invade the outer retina in AMD-like inflammatory scenarios [27, 107], it is likely that the secretion activity of monocytes drives FoxP3 translocation into the nucleus. The cell morphology of the aged RPE implies regenerating activities in response to defects in the monolayer [82]. Thus, IL-1β secreted by monocytes establishes a combined self-regenerating and pro-inflammatory phenotype in the RPE. This would explain why RPE cells in the aged, but healthy retina showed only weak FoxP3 expression in the nucleus since monocytes are lacking in the healthy situation. However, we found that also RPE cells that are in a far distance to laser injuries showed FoxP3 expression, suggesting a spread of information about a dangerous situation over the RPE. This was also observed in cultured ARPE-19 cells after scratching the monolayer, where the information of the injury and the nuclear FoxP3 translocation was spreading over the entire culture. This might be in correlation with a previously published observation of our group that not only the laser site contains activated monocytes and likely microglia cells [75], but there is a pan-retinal activation of microglia that might induce the FoxP3 translocation to the nucleus.

In summary, we found that RPE cells induce FoxP3 expression and nuclear localization under both stress conditions and in pre-degenerative states. FoxP3 expression during RPE cell stress seems essential for cell survival, as suggested by the reduced resistance of ARPE-19 cells to stress after FoxP3-knockout. Cell degeneration or strong local inflammation enhanced FoxP3 phosphorylation and/or translocation into the nucleus, which was also dependent on the cellular status (confluent or not). The consequences of FoxP3 expression and its subcellular localization in RPE cells require further investigation.

## Supplementary Information

**Additional file 1.** The additional file provides statistical comparison of gene expression between wildtype and* Cx3cr1*^*GFP/GFP*^ mice at the different time points, control data for human FoxP3 staining, the growth rate of ARPE-19 to compare with Crisp/Cas treated ARPE-19 cells and uncropped dot plots.

## Data Availability

The datasets used and/or analyzed during the current study are available from the corresponding author on reasonable request.

## Abbreviations

AMD: Age-related macular degeneration
BSA: Bovine serum albumin
DAPI: 4′,6-Diamidine-2-phenylindole
EGTA: Ethylene glycol-bis(aminoethylether)-N,N,N′,N′-tetraacetate
GAPDH: Glycerin aldehyde-3-phosphate-dehydrogenase
GFP: Green fluorescent protein
C3, C3a, C5a: Complement factor 3, -3a, -5
Ca: Calcium
CFH: Complement factor H
CNV: Choroidal neovascularization
CRISPR/Cas9: **C**lustered **R**egularly **I**nterspaced **S**hort **P**alindromic **R**epeats/CRISPR-associated 9
CXCR4: CX-chemokine receptor 4
CX3CR1: CX3-chemokine receptor 1
FoxP3: Forkhead-box-protein P3
IFN-γ: Interferon-γ
IL-1β: Interleukin-1β
MCP-1: Monocyte chemoattractant protein-1
PBS: Phosphate-buffered saline
RPE: Retinal pigment epithelium
TBS: TRIS-buffered saline
TGF-β: Transforming growth factor-β
Treg: Regulatory T cell
VEGF-A: Vascular endothelial growth factor-A
WT: Wild type

## References

[1] Streilein JW (1999). Regional immunity and ocular immune privilege. Chem Immunol.

[2] Streilein JW (1999). Immunoregulatory mechanisms of the eye. Prog Retin Eye Res.

[3] Streilein JW (2003). Ocular immune privilege: therapeutic opportunities from an experiment of nature. Nat Rev Immunol.

[4] Shechter R, London A, Schwartz M (2013). Orchestrated leukocyte recruitment to immune-privileged sites: absolute barriers versus educational gates. Nat Rev Immunol.

[5] Strauss O (2016). Pharmacology of the retinal pigment epithelium, the interface between retina and body system. Eur J Pharmacol.

[6] Strauss O (2005). The retinal pigment epithelium in visual function. Physiol Rev.

[7] Lakkaraju A, Umapathy A, Tan LX, Daniele L, Philp NJ, Boesze-Battaglia K (2020). The cell biology of the retinal pigment epithelium. Prog Retin Eye Res.

[8] Sparrow JR, Hicks D, Hamel CP (2010). The retinal pigment epithelium in health and disease. Curr Mol Med.

[9] Camelo S, Calippe B, Lavalette S, Dominguez E, Hur J, Devevre E (2015). Thinning of the RPE and choroid associated with T lymphocyte recruitment in aged and light-challenged mice. Mol Vis.

[10] Housset M, Sennlaub F (2015). Thrombospondin-1 and pathogenesis of age-related macular degeneration. J Ocul Pharmacol Ther.

[11] Levy O, Calippe B, Lavalette S, Hu SJ, Raoul W, Dominguez E (2015). Apolipoprotein E promotes subretinal mononuclear phagocyte survival and chronic inflammation in age-related macular degeneration. EMBO Mol Med.

[12] Mathis T, Housset M, Eandi C, Beguier F, Touhami S, Reichman S (2017). Activated monocytes resist elimination by retinal pigment epithelium and downregulate their OTX2 expression via TNF-alpha. Aging Cell.

[13] Omri S, Behar-Cohen F, de Kozak Y, Sennlaub F, Verissimo LM, Jonet L (2011). Microglia/macrophages migrate through retinal epithelium barrier by a transcellular route in diabetic retinopathy: role of PKCzeta in the Goto Kakizaki rat model. Am J Pathol.

[14] Touhami S, Beguier F, Augustin S, Charles-Messance H, Vignaud L, Nandrot EF (2018). Chronic exposure to tumor necrosis factor alpha induces retinal pigment epithelium cell dedifferentiation. J Neuroinflammation.

[15] Sennlaub F, Auvynet C, Calippe B, Lavalette S, Poupel L, Hu SJ (2013). CCR2(+) monocytes infiltrate atrophic lesions in age-related macular disease and mediate photoreceptor degeneration in experimental subretinal inflammation in Cx3cr1 deficient mice. EMBO Mol Med.

[16] Busch C, Annamalai B, Abdusalamova K, Reichhart N, Huber C, Lin Y (2017). Anaphylatoxins Activate Ca(2+), Akt/PI3-Kinase, and FOXO1/FoxP3 in the Retinal Pigment Epithelium. Front Immunol.

[17] Brandstetter C, Holz FG, Krohne TU (2015). Complement Component C5a primes retinal pigment epithelial cells for inflammasome activation by lipofuscin-mediated photooxidative damage. J Biol Chem.

[18] Fernandez-Godino R, Garland DL, Pierce EA (2015). A local complement response by RPE causes early-stage macular degeneration. Hum Mol Genet.

[19] Fernandez-Godino R, Pierce EA (2018). C3a triggers formation of sub-retinal pigment epithelium deposits via the ubiquitin proteasome pathway. Sci Rep.

[20] Hu M, Liu B, Jawad S, Ling D, Casady M, Wei L (2011). C5a contributes to intraocular inflammation by affecting retinal pigment epithelial cells and immune cells. Br J Ophthalmol.

[21] Long Q, Cao X, Bian A, Li Y (2016). C3a Increases VEGF and Decreases PEDF mRNA levels in human retinal pigment epithelial cells. Biomed Res Int.

[22] Ramos de Carvalho JE, Klaassen I, Vogels IM, Schipper-Krom S, van Noorden CJ, Reits E (2013). Complement factor C3a alters proteasome function in human RPE cells and in an animal model of age-related RPE degeneration. Invest Ophthalmol Vis Sci.

[23] Schafer N, Grosche A, Schmitt SI, Braunger BM, Pauly D (2017). Complement components showed a time-dependent local expression pattern in constant and acute white light-induced photoreceptor damage. Front Mol Neurosci.

[24] Skeie JM, Fingert JH, Russell SR, Stone EM, Mullins RF (2010). Complement component C5a activates ICAM-1 expression on human choroidal endothelial cells. Invest Ophthalmol Vis Sci.

[25] Vogt SD, Barnum SR, Curcio CA, Read RW (2006). Distribution of complement anaphylatoxin receptors and membrane-bound regulators in normal human retina. Exp Eye Res.

[26] Wang YP, Hui YN, Wang YS, Wang HY (2003). Detection of C5a receptor expressions on human epiretinal membranes and cultured RPE cells by immunohistochemical staining. Xi Bao Yu Fen Zi Mian Yi Xue Za Zhi.

[27] Dietrich L, Lucius R, Roider J, Klettner A (2020). Interaction of inflammatorily activated retinal pigment epithelium with retinal microglia and neuronal cells. Exp Eye Res.

[28] Klettner A, Hamann T, Schluter K, Lucius R, Roider J (2014). Retinal pigment epithelium cells alter the pro-inflammatory response of retinal microglia to TLR-3 stimulation. Acta Ophthalmol.

[29] Kumar MV, Nagineni CN, Chin MS, Hooks JJ, Detrick B (2004). Innate immunity in the retina: Toll-like receptor (TLR) signaling in human retinal pigment epithelial cells. J Neuroimmunol.

[30] Tarallo V, Hirano Y, Gelfand BD, Dridi S, Kerur N, Kim Y (2012). DICER1 loss and Alu RNA induce age-related macular degeneration via the NLRP3 inflammasome and MyD88. Cell.

[31] Terheyden L, Roider J, Klettner A (2021). Basolateral activation with TLR agonists induces polarized cytokine release and reduces barrier function in RPE in vitro. Graefes Arch Clin Exp Ophthalmol.

[32] Zhu Y, Dai B, Li Y, Peng H (2015). C5a and toll-like receptor 4 crosstalk in retinal pigment epithelial cells. Mol Vis.

[33] Sun Q, Gong L, Qi R, Qing W, Zou M, Ke Q (2020). Oxidative stress-induced KLF4 activates inflammatory response through IL17RA and its downstream targets in retinal pigment epithelial cells. Free Radic Biol Med.

[34] Zhang J, Bai Y, Huang L, Qi Y, Zhang Q, Li S (2015). Protective effect of autophagy on human retinal pigment epithelial cells against lipofuscin fluorophore A2E: implications for age-related macular degeneration. Cell Death Dis.

[35] Zamiri P, Masli S, Streilein JW, Taylor AW (2006). Pigment epithelial growth factor suppresses inflammation by modulating macrophage activation. Invest Ophthalmol Vis Sci.

[36] Anderson OA, Finkelstein A, Shima DT (2013). A2E induces IL-1ss production in retinal pigment epithelial cells via the NLRP3 inflammasome. PLoS ONE.

[37] Catalioto RM, Valenti C, Maggi CA, Giuliani S (2015). Enhanced Ca(2+) response and stimulation of prostaglandin release by the bradykinin B2 receptor in human retinal pigment epithelial cells primed with proinflammatory cytokines. Biochem Pharmacol.

[38] Chen M, Hombrebueno JR, Luo C, Penalva R, Zhao J, Colhoun L (2013). Age- and light-dependent development of localised retinal atrophy in CCL2(-/-)CX3CR1(GFP/GFP) mice. PLoS ONE.

[39] Efstathiou NE, Moustafa GA, Maidana DE, Konstantinou EK, Notomi S, Barbisan PRT (2020). Acadesine suppresses TNF-alpha induced complement component 3 (C3), in retinal pigment epithelial (RPE) cells. PLoS ONE.

[40] Huang H, Liu Y, Wang L, Li W (2017). Age-related macular degeneration phenotypes are associated with increased tumor necrosis-alpha and subretinal immune cells in aged Cxcr5 knockout mice. PLoS ONE.

[41] Jasielska M, Semkova I, Shi X, Schmidt K, Karagiannis D, Kokkinou D (2010). Differential role of tumor necrosis factor (TNF)-alpha receptors in the development of choroidal neovascularization. Invest Ophthalmol Vis Sci.

[42] Li W, Ma N, Liu MX, Ye BJ, Li YJ, Hu HY (2019). C1q/TNF-related protein-9 attenuates retinal inflammation and protects blood-retinal barrier in db/db mice. Eur J Pharmacol.

[43] van Bilsen K, van Hagen PM, Bastiaans J, van Meurs JC, Missotten T, Kuijpers RW (2011). The neonatal Fc receptor is expressed by human retinal pigment epithelial cells and is downregulated by tumour necrosis factor-alpha. Br J Ophthalmol.

[44] Zhang SX, Wang JJ, Gao G, Shao C, Mott R, Ma JX (2006). Pigment epithelium-derived factor (PEDF) is an endogenous antiinflammatory factor. FASEB J.

[45] Ando Y, Sato Y, Kudo A, Watanabe T, Hirakata A, Okada AA (2020). Antiinflammatory effects of the NFkappaB inhibitor dehydroxymethylepoxyquinomicin on ARPE19 cells. Mol Med Rep.

[46] Ehlken C, Grundel B, Michels D, Junker B, Stahl A, Schlunck G (2015). Increased expression of angiogenic and inflammatory proteins in the vitreous of patients with ischemic central retinal vein occlusion. PLoS ONE.

[47] Jo DH, Yun JH, Cho CS, Kim JH, Kim JH, Cho CH (2019). Interaction between microglia and retinal pigment epithelial cells determines the integrity of outer blood-retinal barrier in diabetic retinopathy. Glia.

[48] Lin T, Walker GB, Kurji K, Fang E, Law G, Prasad SS (2013). Parainflammation associated with advanced glycation endproduct stimulation of RPE in vitro: implications for age-related degenerative diseases of the eye. Cytokine.

[49] Diedrichs-Möhring M, Niesik S, Priglinger CS, Thurau SR, Obermayr F, Sperl S (2018). Intraocular DHODH-inhibitor PP-001 suppresses relapsing experimental uveitis and cytokine production of human lymphocytes, but not of RPE cells. J Neuroinflammation.

[50] Klettner A, Brinkmann A, Winkelmann K, Kackenmeister T, Hildebrandt J, Roider J (2020). Effect of long-term inflammation on viability and function of RPE cells. Exp Eye Res.

[51] Amadi-Obi A, Yu CR, Dambuza I, Kim SH, Marrero B, Egwuagu CE (2012). Interleukin 27 induces the expression of complement factor H (CFH) in the retina. PLoS ONE.

[52] Marazita MC, Dugour A, Marquioni-Ramella MD, Figueroa JM, Suburo AM (2016). Oxidative stress-induced premature senescence dysregulates VEGF and CFH expression in retinal pigment epithelial cells: implications for age-related macular degeneration. Redox Biol.

[53] Pauly D, Agarwal D, Dana N, Schafer N, Biber J, Wunderlich KA (2019). Cell-type-specific complement expression in the healthy and diseased retina. Cell Rep.

[54] Weinberger AW, Eddahabi C, Carstesen D, Zipfel PF, Walter P, Skerka C (2014). Human complement factor H and factor H-like protein 1 are expressed in human retinal pigment epithelial cells. Ophthalmic Res.

[55] Zhang Y, Huang Q, Tang M, Zhang J, Fan W (2015). Complement Factor H expressed by retinal pigment epithelium cells can suppress neovascularization of human umbilical vein endothelial cells: an in vitro study. PLoS ONE.

[56] Mulfaul K, Ozaki E, Fernando N, Brennan K, Chirco KR, Connolly E (2020). Toll-like receptor 2 facilitates oxidative damage-induced retinal degeneration. Cell Rep.

[57] Luo C, Zhao J, Madden A, Chen M, Xu H (2013). Complement expression in retinal pigment epithelial cells is modulated by activated macrophages. Exp Eye Res.

[58] Rohrer B, Coughlin B, Kunchithapautham K, Long Q, Tomlinson S, Takahashi K (2011). The alternative pathway is required, but not alone sufficient, for retinal pathology in mouse laser-induced choroidal neovascularization. Mol Immunol.

[59] Trakkides TO, Schafer N, Reichenthaler M, Kuhn K, Brandwijk R, Toonen EJM (2019). Oxidative Stress increases endogenous complement-dependent inflammatory and angiogenic responses in retinal pigment epithelial cells independently of exogenous complement sources. Antioxidants (Basel)..

[60] Hori S, Nomura T, Sakaguchi S (2003). Control of regulatory T cell development by the transcription factor Foxp3. Science.

[61] Kasprowicz DJ, Smallwood PS, Tyznik AJ, Ziegler SF (2003). Scurfin (FoxP3) controls T-dependent immune responses in vivo through regulation of CD4+ T cell effector function. J Immunol.

[62] Wang J, Ioan-Facsinay A, Voort E, Huizinga T, Toes R (2007). Transient expression of FOXP3 in human activated nonregulatory CD4+ T cells. Eur J Immunol.

[63] Wildner G (2019). Are rats more human than mice?. Immunobiology.

[64] Magg T, Mannert J, Ellwart JW, Schmid I, Albert MH (2012). Subcellular localization of FOXP3 in human regulatory and nonregulatory T cells. Eur J Immunol.

[65] Dong Y, Yang C, Pan F (2021). Post-translational regulations of Foxp3 in treg cells and their therapeutic applications. Front Immunol.

[66] Munoz-Rojas AR, Mathis D (2021). Tissue regulatory T cells: regulatory chameleons. Nat Rev Immunol.

[67] Georgiev P, Charbonnier LM, Chatila TA (2019). Regulatory T Cells: the Many Faces of Foxp3. J Clin Immunol.

[68] Wang L, Liu R, Li W, Chen C, Katoh H, Chen GY (2009). Somatic single hits inactivate the X-linked tumor suppressor FOXP3 in the prostate. Cancer Cell.

[69] Zhang HY, Sun H (2010). Up-regulation of Foxp3 inhibits cell proliferation, migration and invasion in epithelial ovarian cancer. Cancer Lett.

[70] Zuo T, Wang L, Morrison C, Chang X, Zhang H, Li W (2007). FOXP3 is an X-linked breast cancer suppressor gene and an important repressor of the HER-2/ErbB2 oncogene. Cell.

[71] Redpath M, Xu B, van Kempen LC, Spatz A (2011). The dual role of the X-linked FoxP3 gene in human cancers. Mol Oncol.

[72] Tallon B, Bhawan J (2010). FoxP3 expression is increased in cutaneous squamous cell carcinoma with perineural invasion. J Cutan Pathol.

[73] Tan B, Behren A, Anaka M, Vella L, Cebon J, Mariadason JM (2012). FOXP3 is not mutated in human melanoma. Pigment Cell Melanoma Res.

[74] Combadiere C, Feumi C, Raoul W, Keller N, Rodero M, Pezard A (2007). CX3CR1-dependent subretinal microglia cell accumulation is associated with cardinal features of age-related macular degeneration. J Clin Invest.

[75] Crespo-Garcia S, Corkhill C, Roubeix C, Davids AM, Kociok N, Strauss O (2017). Inhibition of Placenta Growth Factor Reduces Subretinal Mononuclear Phagocyte Accumulation in Choroidal Neovascularization. Invest Ophthalmol Vis Sci.

[76] Lingeman E, Jeans C, Corn JE (2017). Production of Purified CasRNPs for Efficacious Genome Editing. Curr Protoc Mol Biol.

[77] Hultquist JF, Schumann K, Woo JM, Manganaro L, McGregor MJ, Doudna J (2016). A Cas9 ribonucleoprotein platform for functional genetic studies of HIV-host interactions in primary human T Cells. Cell Rep.

[78] Schumann K, Raju SS, Lauber M, Kolb S, Shifrut E, Cortez JT (2020). Functional CRISPR dissection of gene networks controlling human regulatory T cell identity. Nat Immunol.

[79] Brinkman EK, Chen T, Amendola M, van Steensel B (2014). Easy quantitative assessment of genome editing by sequence trace decomposition. Nucleic Acids Res.

[80] Schneider CA, Rasband WS, Eliceiri KW (2012). NIH Image to ImageJ: 25 years of image analysis. Nat Methods.

[81] Busch C, Annamalai B, Abdusalamova K, Reichhart N, Huber C, Lin Y (2017). Anaphylatoxins Activate Ca2+, Akt/PI3-Kinase, and FOXO1/FoxP3 in the retinal pigment epithelium. Front Immunol.

[82] Chen M, Rajapakse D, Fraczek M, Luo C, Forrester JV, Xu H (2016). Retinal pigment epithelial cell multinucleation in the aging eye - a mechanism to repair damage and maintain homoeostasis. Aging Cell.

[83] Bozic CR, Gerard NP, von Uexkull-Guldenband C, Kolakowski LF, Conklyn MJ, Breslow R (1994). The murine interleukin 8 type B receptor homologue and its ligands. Expression and biological characterization. J Biol Chem.

[84] Rovai LE, Herschman HR, Smith JB (1998). The murine neutrophil-chemoattractant chemokines LIX, KC, and MIP-2 have distinct induction kinetics, tissue distributions, and tissue-specific sensitivities to glucocorticoid regulation in endotoxemia. J Leukoc Biol.

[85] Diedrichs-Mohring M, Nelson PJ, Proudfoot AE, Thurau SR, Wildner G (2005). The effect of the CC chemokine receptor antagonist Met-RANTES on experimental autoimmune uveitis and oral tolerance. J Neuroimmunol.

[86] Thurau SR, Mempel TR, Flugel A, Diedrichs-Mohring M, Krombach F, Kawakami N (2004). The fate of autoreactive, GFP+ T cells in rat models of uveitis analyzed by intravital fluorescence microscopy and FACS. Int Immunol.

[87] Obert E, Strauss R, Brandon C, Grek C, Ghatnekar G, Gourdie R (2017). Targeting the tight junction protein, zonula occludens-1, with the connexin43 mimetic peptide, alphaCT1, reduces VEGF-dependent RPE pathophysiology. J Mol Med (Berl).

[88] Woodell A, Coughlin B, Kunchithapautham K, Casey S, Williamson T, Ferrell WD (2013). Alternative complement pathway deficiency ameliorates chronic smoke-induced functional and morphological ocular injury. PLoS ONE.

[89] Tian J, Ishibashi K, Honda S, Boylan SA, Hjelmeland LM, Handa JT (2005). The expression of native and cultured human retinal pigment epithelial cells grown in different culture conditions. Br J Ophthalmol.

[90] Samuel W, Jaworski C, Postnikova OA, Kutty RK, Duncan T, Tan LX (2017). Appropriately differentiated ARPE-19 cells regain phenotype and gene expression profiles similar to those of native RPE cells. Mol Vis.

[91] Schumann K, Lin S, Boyer E, Simeonov DR, Subramaniam M, Gate RE (2015). Generation of knock-in primary human T cells using Cas9 ribonucleoproteins. Proc Natl Acad Sci U S A.

[92] Kim H, Kim JS (2014). A guide to genome engineering with programmable nucleases. Nat Rev Genet.

[93] Wooff Y, Man SM, Aggio-Bruce R, Natoli R, Fernando N. IL-1 Family Members Mediate Cell Death, Inflammation and Angiogenesis in Retinal Degenerative Diseases. Frontiers in Immunology. 2019;10. 10.3389/fimmu.2019.01618 PMC6646526 31379825

[94] Dabouz R, Cheng CWH, Abram P, Omri S, Cagnone G, Sawmy KV (2020). An allosteric interleukin-1 receptor modulator mitigates inflammation and photoreceptor toxicity in a model of retinal degeneration. J Neuroinflammation.

[95] Zanzottera EC, Ach T, Huisingh C, Messinger JD, Spaide RF, Curcio CA (2016). Visualizing retinal pigment epithelium phenotypes in the transition to geographic atrophy in age-related macular degeneration. Retina.

[96] Imai A, Sugita S, Kawazoe Y, Horie S, Yamada Y, Keino H (2012). Immunosuppressive properties of regulatory T cells generated by incubation of peripheral blood mononuclear cells with supernatants of human RPE cells. Invest Ophthalmol Vis Sci.

[97] Vega JL, Saban D, Carrier Y, Masli S, Weiner HL (2010). Retinal pigment epithelial cells induce foxp3(+) regulatory T cells via membrane-bound TGF-beta. Ocul Immunol Inflamm.

[98] Tran DQ (2012). TGF-beta: the sword, the wand, and the shield of FOXP3(+) regulatory T cells. J Mol Cell Biol.

[99] Allan SE, Crome SQ, Crellin NK, Passerini L, Steiner TS, Bacchetta R (2007). Activation-induced FOXP3 in human T effector cells does not suppress proliferation or cytokine production. Int Immunol.

[100] Gavin MA, Torgerson TR, Houston E, DeRoos P, Ho WY, Stray-Pedersen A (2006). Single-cell analysis of normal and FOXP3-mutant human T cells: FOXP3 expression without regulatory T cell development. Proc Natl Acad Sci U S A.

[101] Kmieciak M, Gowda M, Graham L, Godder K, Bear HD, Marincola FM (2009). Human T cells express CD25 and Foxp3 upon activation and exhibit effector/memory phenotypes without any regulatory/suppressor function. J Transl Med.

[102] Pillai V, Ortega SB, Wang CK, Karandikar NJ (2007). Transient regulatory T-cells: a state attained by all activated human T-cells. Clin Immunol.

[103] Wang J, Ioan-Facsinay A, van der Voort EI, Huizinga TW, Toes RE (2007). Transient expression of FOXP3 in human activated nonregulatory CD4+ T cells. Eur J Immunol.

[104] Deng G, Song X, Fujimoto S, Piccirillo CA, Nagai Y, Greene MI (2019). Foxp3 Post-translational Modifications and Treg Suppressive Activity. Front Immunol.

[105] Nie H, Zheng Y, Li R, Guo TB, He D, Fang L (2013). Phosphorylation of FOXP3 controls regulatory T cell function and is inhibited by TNF-alpha in rheumatoid arthritis. Nat Med.

[106] Jaffe GJ, Van Le L, Valea F, Haskill S, Roberts W, Arend WP (1992). Expression of interleukin-1 alpha, interleukin-1 beta, and an interleukin-1 receptor antagonist in human retinal pigment epithelial cells. Exp Eye Res.

[107] Lavalette S, Raoul W, Houssier M, Camelo S, Levy O, Calippe B (2011). Interleukin-1beta inhibition prevents choroidal neovascularization and does not exacerbate photoreceptor degeneration. Am J Pathol.

[108] Beskina O, Miller A, Mazzocco-Spezzia A, Pulina MV, Golovina VA (2007). Mechanisms of interleukin-1beta-induced Ca2+ signals in mouse cortical astrocytes: roles of store- and receptor-operated Ca2+ entry. Am J Physiol Cell Physiol.

[109] Lucas K, Karamichos D, Mathew R, Zieske JD, Stein-Streilein J (2012). Retinal laser burn-induced neuropathy leads to substance P-dependent loss of ocular immune privilege. J Immunol.

[110] Rossi O, Karczewski J, Stolte EH, Brummer RJ, van Nieuwenhoven MA, Meijerink M (2013). Vectorial secretion of interleukin-8 mediates autocrine signalling in intestinal epithelial cells via apically located CXCR1. BMC Res Notes.

