# Supplementary material for: FoxP3 expression by retinal pigment epithelial cells: transcription factor with potential relevance for the pathology of age-related macular degeneration
Source: J Neuroinflammation. 2022 Oct 22;19:260. doi: 10.1186/s12974-022-02620-w (PMC9588251; doi:10.1186/s12974-022-02620-w)
Supplement: Supplementary file 1 — Additional file 1. The additional file provides statistical comparison of gene expression between wildtype and Cx3cr1GFP/GFP mice at the different time points, control data for human FoxP3 staining, the growth rate of ARPE-19 to compare with Crisp/Cas treated ARPE-19 cells and uncropped dot plots. [file 12974_2022_2620_MOESM1_ESM.docx]

**Supplemental Data:**

**1. Comparison of cytokine mRNA expression in WT mice with *Cx3cr1^GFP/GFP^* mice at the time points 8 months and 12 months**

**
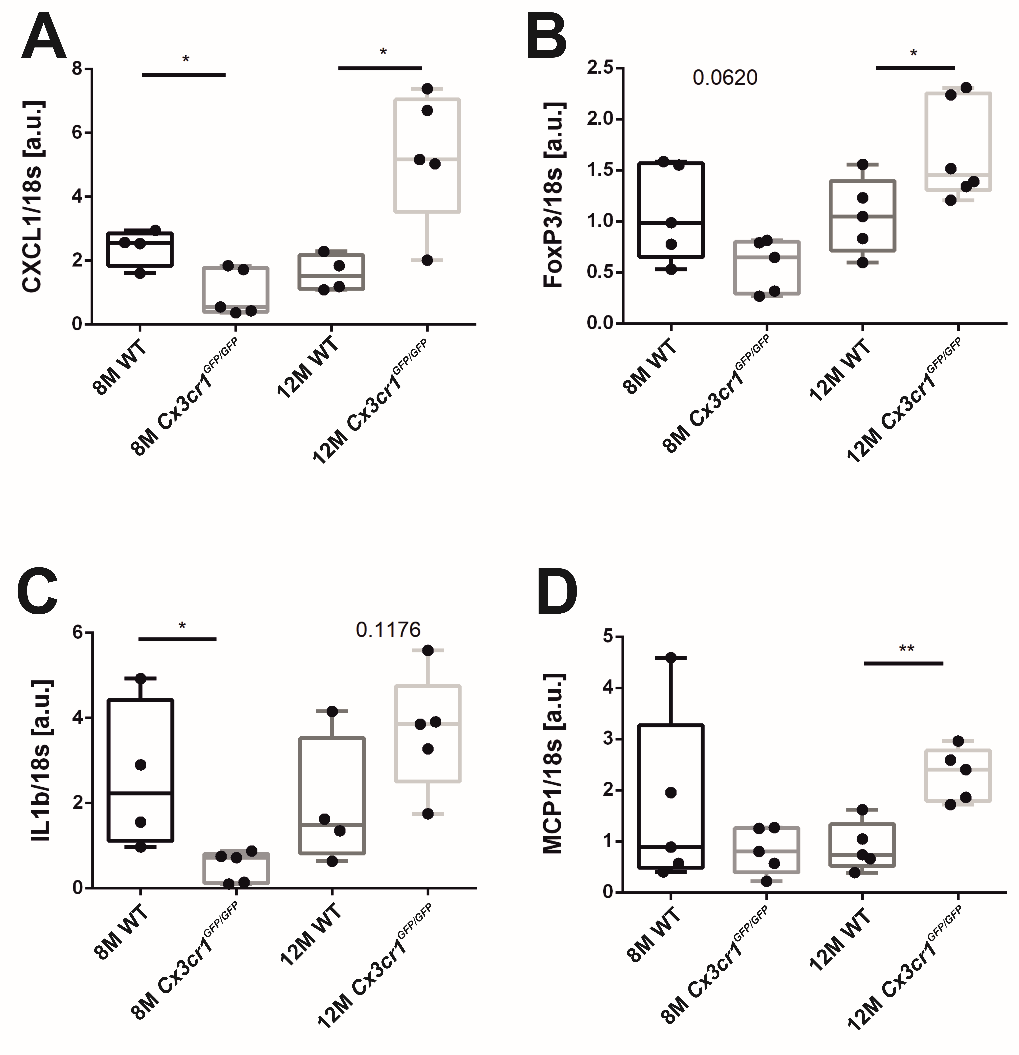
**

**Suppl. Figure 1:** The expression of the cytokines Cxcl1 (**A**), FoxP3 (**B**), IL-1β (**C**) and MCP-1 (**D**) was increased in RPE of *Cx3cr1^GFP/GFP^* mice at the age of 12 months compared to wildtype at the same age. Interestingly, at the age of 8 months the expression of Cxcl1, MCP-1 and IL-1β appeared slightly decreased in the *Cx3cr1^GFP/GFP^* mice compared to the wildtype animals at the same age. We explain this by an earlier interaction of the RPE with monocytes in *Cx3cr1^GFP/GFP^*, which might result in an anti-inflammatory reaction of the RPE. (mean ± SEM; , p values *p < 0.05, **p < 0.01 n = 3).

**2. Controls for FoxP3 antibody staining**

**A. Secondary antibody only and FastRed (left panel); FastRed with anti-RPE65 antibody (right panel)**

**
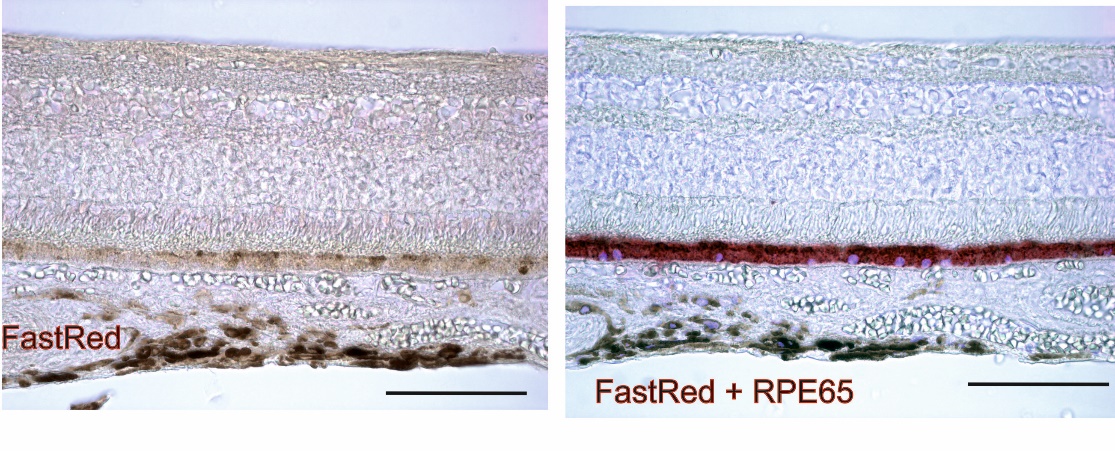
**

**B. Positive control: T cells in lymph node**

**
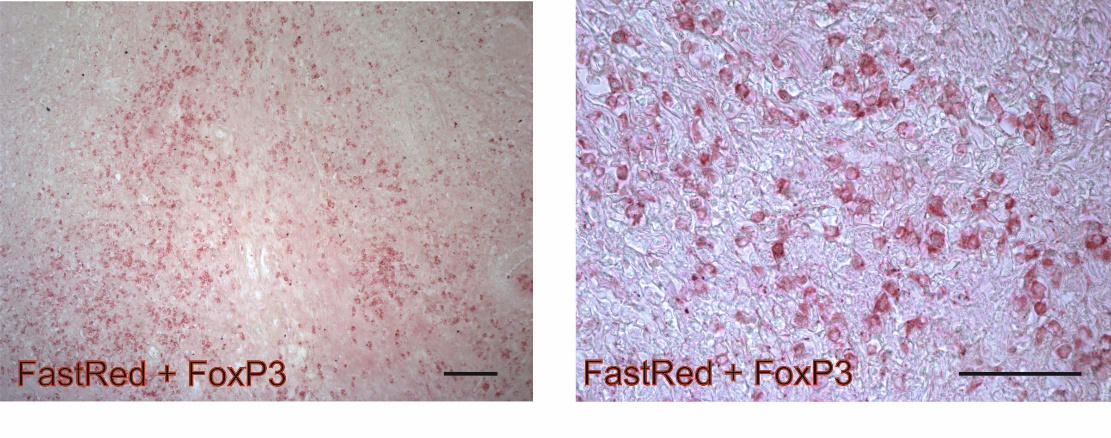
**

**Suppl. Figure 2:** negative and positive control for FoxP3 staining in human tissues using FastRed as substrate: **A:** negative control using sagittal sections of human retina: left panel: FastRed only does not stain the retina; right panel: staining the human retina with an antibody against the RPE specific protein RPE65 results in a specific staining of the RPE (substrate: FastRed). **B:** positive control; FoxP3 staining of Treg cells in the human lymph node; left panel overview and right panel at higher magnification. (size bars: overview: 60 µm; higher magnifications: 30 µm)

**3. Growth rate of non-treated ARPE-19 cells**





**Suppl. Figure 3: Growth rate of untreated ARPE-19 cells:** ARPE-19 cells were seeded in the same density as after CRISPR/Cas9 treatment; cell number was measured after 6 days. N = 4.

**4. Uncropped dot blots:**

**
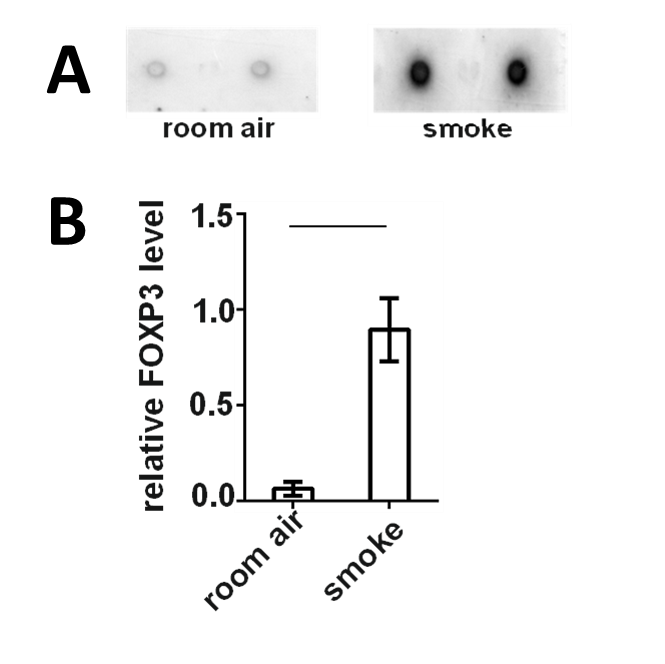
A. Uncropped dot blots used for Figure 4**


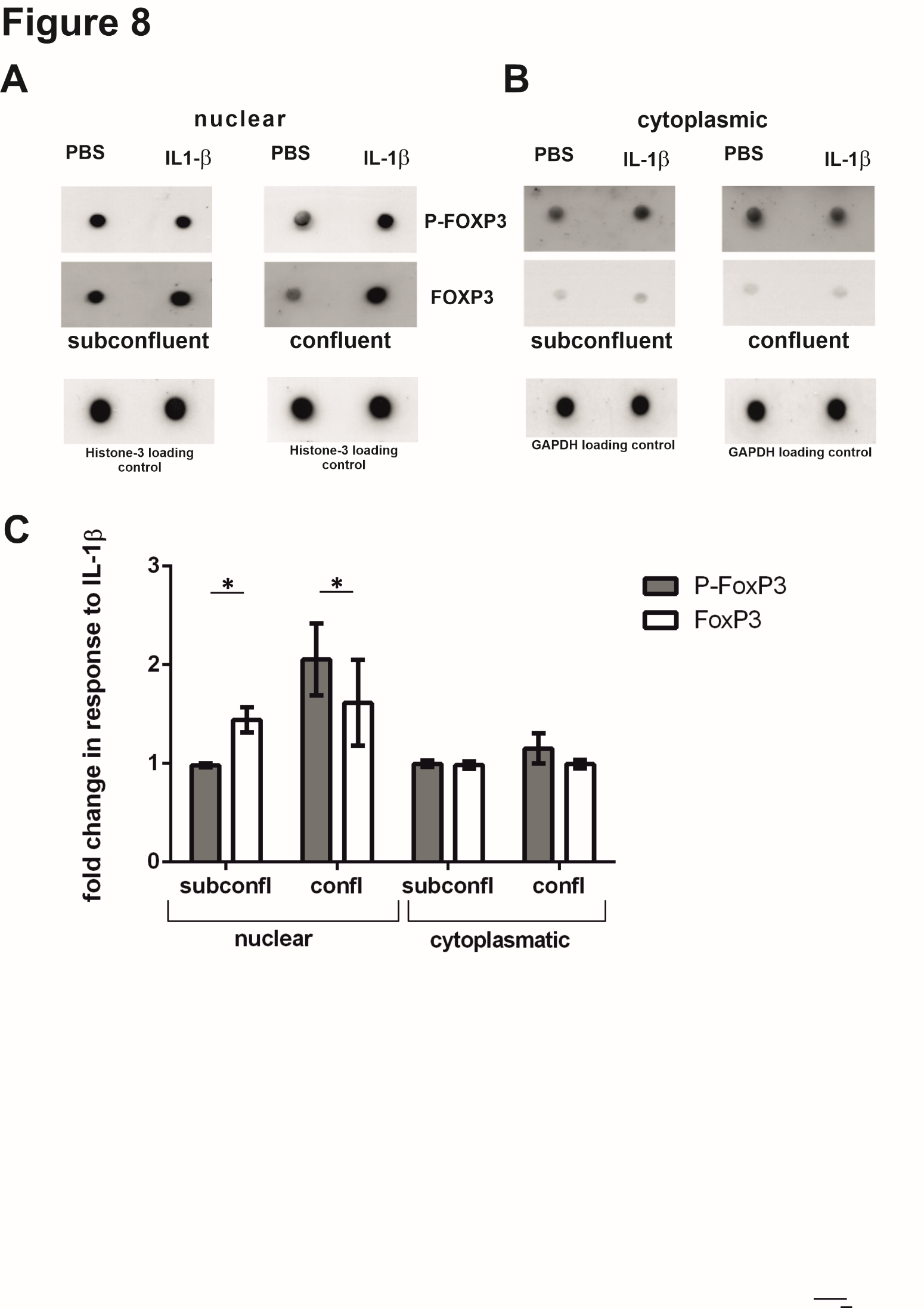


**B. Uncropped dot blots used for Figure 8**

**Suppl. Figure 4: Uncropped dot blots:** A. Original dot blots of phospho-FoxP3 from the smoke exposure experiments (figure 4). B. original dot plots of FoxP3 and phospho-FoxP3 from confluent or subconfluent ARPE-19 cultures; cytoplasmic and nuclear fractions of FoxP3 with or without IL-1β (Figure 8).
